# Physiological, Biochemical, and Transcriptional Responses to Single and Combined Abiotic Stress in Stress-Tolerant and Stress-Sensitive Potato Genotypes

**DOI:** 10.3389/fpls.2020.00169

**Published:** 2020-02-27

**Authors:** Ufuk Demirel, Wayne L. Morris, Laurence J. M. Ducreux, Caner Yavuz, Arslan Asim, Ilknur Tindas, Raymond Campbell, Jenny A. Morris, Susan R. Verrall, Pete E. Hedley, Zahide N. O. Gokce, Sevgi Caliskan, Emre Aksoy, Mehmet E. Caliskan, Mark A. Taylor, Robert D. Hancock

**Affiliations:** ^1^ Faculty of Agricultural Sciences and Technologies, Niğde Ömer Halisdemir University, Niğde, Turkey; ^2^ Cell and Molecular Sciences, The James Hutton Institute, Dundee, United Kingdom; ^3^ Information and Computational Sciences, The James Hutton Institute, Dundee, United Kingdom

**Keywords:** abiotic stress, transcriptome, metabolome, crop physiology, crop resilience

## Abstract

Potato production is often constrained by abiotic stresses such as drought and high temperatures which are often present in combination. In the present work, we aimed to identify key mechanisms and processes underlying single and combined abiotic stress tolerance by comparative analysis of tolerant and susceptible cultivars. Physiological data indicated that the cultivars Desiree and Unica were stress tolerant while Agria and Russett Burbank were stress susceptible. Abiotic stress caused a greater reduction of photosynthetic carbon assimilation in the susceptible cultivars which was associated with a lower leaf transpiration rate. Oxidative stress, as estimated by the accumulation of malondialdehyde was not induced by stress treatments in any of the genotypes with the exception of drought stress in Russett Burbank. Stress treatment resulted in increases in ascorbate peroxidase activity in all cultivars except Agria which increased catalase activity in response to stress. Transcript profiling highlighted a decrease in the abundance of transcripts encoding proteins associated with PSII light harvesting complex in stress tolerant cultivars. Furthermore, stress tolerant cultivars accumulated fewer transcripts encoding a type-1 metacaspase implicated in programmed cell death. Stress tolerant cultivars exhibited stronger expression of genes associated with plant growth and development, hormone metabolism and primary and secondary metabolism than stress susceptible cultivars. Metabolite profiling revealed accumulation of proline in all genotypes following drought stress that was partially suppressed in combined heat and drought. On the contrary, the sugar alcohols inositol and mannitol were strongly accumulated under heat and combined heat and drought stress while galactinol was most strongly accumulated under drought. Combined heat and drought also resulted in the accumulation of Valine, isoleucine, and lysine in all genotypes. These data indicate that single and multiple abiotic stress tolerance in potato is associated with a maintenance of CO_2_ assimilation and protection of PSII by a reduction of light harvesting capacity. The data further suggests that stress tolerant cultivars suppress cell death and maintain growth and development *via* fine tuning of hormone signaling, and primary and secondary metabolism. This study highlights potential targets for the development of stress tolerant potato cultivars.

## Introduction

Potato (*Solanum tuberosum* L.) is one of the most important food crops in the world where it ranks only behind rice and wheat in terms of global production (Birch et al., 2012). The crop is particularly vulnerable to elevated temperature which results in significant reductions in tuber yields (Gregory, 1965; Slater, 1968). For example, soil temperature higher than 18°C reduces tuber yield, especially when combined with high ambient air temperature. Furthermore, an estimated average temperature increase of 1–1.4°C in current potato growing regions by 2040 is predicted to reduce global potential yield by 18 to 32% (Hijmans, 2003). Such yield reductions are in part driven by the temperature sensitivity of carbon transport to sink organs leading to reduced incorporation of assimilated carbon into starch in the tuber (Wolf et al., 1991; Hancock et al., 2014). Moreover, excessive temperatures have a detrimental effect on photosynthetic performance including severe inhibition of CO_2_ fixation and chlorophyll loss in sensitive species (Reynolds et al., 1990). Recent work has demonstrated the mechanisms by which tuberization signaling and initiation is impaired at elevated temperature (Lehretz et al., 2019; Morris et al., 2019). However, there is wide variation for heat stress tolerance across potato germplasm that could be exploited to ensure the sustainability of yield in warmer climates (Levy and Veilleux, 2007; Trapero-Mozos et al., 2018a).

Water limitation also has a negative impact on potato development resulting in decreased tuber yield in areas with inconsistent rainfall or poor irrigation (Evers et al., 2010; Thiele et al., 2010; Monneveux et al., 2013). Most of the potato varieties currently grown are susceptible to drought stress mainly due to shallow root systems and a lack of recovery following water stress (Iwama and Yamaguchi, 2006). The extent to which drought effects potato tuber yield is dependent on the timing, duration, and severity of the stress (Jeffery, 1995) where plant emergence and onset of tuberization are considered the most critical periods when water stress affects tuber yield (Martínez and Moreno, 1992; Obidiegwu et al., 2015). Effects of drought include a decrease in plant growth (Deblonde and Ledent, 2001) and reduction in the number and size of tubers (Eiasum et al., 2007; Schafleitner et al., 2007). Tuber yield under limited water conditions is influenced by a combination of morphological and physiological processes including, photosynthesis, leaf expansion, and senescence, assimilate partitioning, tuber initiation, and tuber bulking (van Loon, 1981). Like heat stress, drought may also affect tuber quality including increased accumulation of toxic glycoalkaloids (Bejarano et al., 2000) and tuber defects such as cracking, secondary growth, malformations, hollow heart, and internal brown spot (Harris, 1978). One of the major causes of damage and subsequent reduction in yield of agricultural crops resulting from abiotic stress is the overproduction of reactive oxygen species (ROS) (You and Chan, 2015). High concentrations of ROS, including hydrogen peroxide (H_2_O_2_), superoxide anions (O_2_•^−^), hydroxyl radical (OH•), and singlet oxygen (^1^O_2_), can trigger a multitude of detrimental responses which include lipid peroxidation of cellular membranes, breakdown of photosynthetic pigments, denaturation of proteins, carbohydrate oxidation, DNA damage, and decreased enzyme activities (Noctor and Foyer, 1998). However, ROS are also important signaling molecules providing cues to allow plants to adjust their metabolism to the prevailing environment (Hancock, 2017) therefore, the ability of crop plants to regulate ROS homeostasis is of vital importance to ensure survival under unfavorable environmental conditions.

In a changing global climate, fluctuations in temperature and precipitation are likely to increase (Intergovernmental Panel on Climate Change 2014 report; http://www.ipcc.ch). Temperature and water stresses often occur together leading to decreased yield and quality losses. Many studies have investigated the response of plants to a combination of different abiotic stresses (for a recent review see Zandalinas et al., 2018) including one report for potato (Rensink et al., 2005). These studies have concluded that combinations of stresses impose a specific set of plant responses that cannot be predicted from the results of studies on plants subjected to a single abiotic stress. Furthermore, targeted breeding approaches are hampered by a lack of fundamental knowledge of how plants perceive and respond to the specific combinations of abiotic stresses experienced in the growing environment. There is therefore a clear need to understand the responses to combined abiotic stresses under real-world conditions, to elucidate molecular mechanisms by which crops can maintain yield and quality in the face of abiotic stress and to define markers that can be efficiently applied in breeding to generate potato varieties that are more tolerant to combined abiotic stress.

An initial objective of this study was to determine the abiotic stress conditions that enabled discrimination between stress susceptible and tolerant genotypes based upon physiological and biochemical responses. Furthermore, we wished to determine whether genotypes that were previously reported to be tolerant to drought or heat stress alone also exhibited tolerance to a combined heat and drought stress. This enabled the rational design of experiments that aimed to identify key transcripts, metabolites, and biological processes that were consistently associated with stress tolerance to inform the future development of stress tolerant potato varieties.

## Materials and Methods

### Plant Material

The four potato genotypes used in this study were selected based on their previously reported abiotic stress tolerance. Desiree and Unica are classed as both heat and drought tolerant (Basu and Minhas, 1991; www.europotato.org; Gutiérrez-Rosales et al., 2007; Rolando et al., 2015) whereas Russet Burbank and Agria are considered heat and drought sensitive (Ahn et al., 2004; Stark et al., 2013; Demirel et al., 2017).

### Growth Conditions and Stress Treatments

Seed tubers were planted in 5 L pots containing compost and perlite (2:1), and plants were grown in environmentally controlled walk-in chambers with a 14 h photoperiod, 24°C day/18°C night temperature, and 60–70% relative humidity until stress treatment. All plants were irrigated regularly until the beginning of stress application, and fertilizer N-P-K (18%-18%-18%) was applied twice to all plants after emergence. Stress treatments were initiated 27 days after emergence by dividing plants randomly into four groups as control, drought, heat, and combined drought-heat treatments. The experiment was carried out with three replications, and each replication consisted of two pots each containing two potato plants. Two identical growth chambers were used for stress treatments, one for control and drought, the another for heat and combined drought and heat. Control plants were grown under optimum conditions as described above until samples were harvested. Drought was applied to plants by withdrawing irrigation for 12 days at 24/18°C (day/night). For heat treatment, plants were exposed to a gradual temperature increase in both day and night for 9 days, until the temperature reached to 39/27°C, then, a constant high temperature of 39/27°C was applied for 3 days (**Supplementary Figure S1**). In both control and heat treatments, relative humidity was maintained at 60–70% throughout treatment. For heat treatment alone, plants continued to be irrigated throughout treatment whereas for combined drought and heat treatment irrigation was withdrawn for the entire period. Physiological traits such as relative water content (RWC), chlorophyll index (SPAD), leaf temperature, and photosynthetic traits were measured directly on the 12th day of stress application. After measurement of the physiological traits, fully expanded upper third and fourth leaves were immediately harvested from the three replicates, separately. Part of the leaf samples were immediately frozen in liquid nitrogen then stored at −80°C for RNA extraction and biochemical and antioxidant enzyme activity assays. A separate sub-sample of the leaf material was freeze-dried and stored at room temperature for metabolite profiling.

### Quantification of Physiological Traits

#### Relative Water Content

Twelve days after initiation of stress treatment, RWC was measured on fully expanded upper third or fourth leaves. Three biological replicates were collected from separate plants. The fresh weight of harvested leaflets was immediately measured prior to incubation in distilled water overnight at room temperature. Excess water was removed by blotting with tissue paper and the turgid weight was recorded. Turgid leaf samples were first dried in a microwave oven at 500 W power for 10 min and then in a drying oven at 95°C for 2–3 h. Finally, dried leaf sample weights were recorded. RWC values of genotypes were calculated using the following equation.

RWC (%): [(Fresh weight–Dry weight)/(Turgid weight–Dry weight)]∗100

#### Chlorophyll Content

Chlorophyll content was estimated on fully expanded upper leaves using a chlorophyll meter (Minolta SPAD 502, USA). Chlorophyll measurements were performed on five leaves of two individual plants and the average value was considered as one biological replicate. Three biological replicates were used to estimate mean and standard error. Chlorophyll measurement was performed on the 12th day of stress treatment.

#### Leaf Temperature

Leaf temperature was measured on fully expanded upper leaves using an infrared thermometer (Sinometer BM380, China). Leaf temperature measurements were on three leaves of two individual plants and the average value was considered as one biological replicate. Three biological replicates were used to estimate mean and standard error. The leaf temperature measurement was performed on the 12th day of stress treatment.

#### Leaf Gas Exchange

Photosynthetic rate (Pn), transpiration rate (E), and stomatal conductance (Gs) were measured on fully expanded upper third or fourth leaves of genotypes with a LICOR LI-6400XT portable photosynthesis system using a built-in light source. Photosynthetically active radiation (PAR) was maintained at 1,500 μmol m^–2^s^–1^ and CO_2_ concentration was maintained at 400 μmol mol^−1^. Measurements of Pn, E, and Gs for each genotype were performed on one plant from each of three biological replicates. The Pn, E, and Gs measurements were taken periodically up until the 12th day of stress treatment.

### Biochemical Assays

#### Malondialdehyde Content

On the 12th day of stress treatment, the level of lipid peroxidation was quantified by measuring the amount of malondialdehyde (MDA) as determined by the thiobarbituric acid (TBA) reaction described by Heath and Packer (1968). Two upper fully expanded leaves were separately analyzed from each plant and the average value considered to be one biological replicate. Three biological replicates were used to estimate mean values and standard error. Briefly, leaf samples (0.2–0.3 g) were homogenized in 2 ml of 0.1% (w/v) trichloroacetic acid (TCA) and the homogenates were centrifuged at 10,000 × g for 20 min. After centrifugation, 2 ml of 20% (w/v) TCA containing 0.5% (w/v) TBA were added to 1.8 ml of the supernatants. After incubation in boiling water for 30 min, the mixture was quickly cooled in an ice bath for 10 min. The samples were aliquoted into two separate 2 ml tubes, and centrifuged at 10,000 × g for 5 min. Finally, the absorbance of the supernatant was measured at 532 nm. The solution containing 0.5% TBA and 20% TCA was used as a blank. To calculate MDA content, the value for nonspecific absorption at 600 nm was subtracted from the readings at 532 nm and an extinction coefficient of 155 mM^−1^ cm^−1^ was used. The values for MDA content are expressed as µmol/g fresh weight (FW).

$$\text{MDA }\left(\text{µmol/g FW}\right)=\left[\left(\text{A}532-\text{A}600\right)/155\right]\text{x}10^{3}\text{x dilution factor x }\left(1\text{/tissue weight g}\right)$$

#### Proline Content

On 12th day of stress treatment, proline content was determined according to the method of Bates et al. (1973) with minor modifications. Briefly, leaf samples (0.2–0.3 g) were ground in 2 ml of 3% sulfosalicylic acid and then centrifuged at 10,000 × g for 20 min at 4 °C. The supernatants (1 ml) were mixed with 1 ml of freshly prepared acid–ninhydrin solution (1.25 g of ninhydrin, 30 ml of glacial acetic acid, 20 ml of 6 M orthophosphoric acid). The mix was shaken gently 3–4 times and incubated in boiling water for 1 h. Then, the reaction was terminated by placing the samples on ice for 10 min. A 2 ml aliquot of toluene was added to the reaction mixtures and vortexed for 15 s. The tubes were left undisturbed for 1 h at room temperature in the dark to allow the separation of solvent and aqueous phases. The toluene phase was then carefully collected and the absorbance values were measured at 520 nm. Toluene was used as a blank. The concentration of proline was estimated from leaves harvested from three independent plants by calibration against a standard curve.

### Antioxidant Enzyme Assays

For enzyme extraction from the plants, 200 mg of frozen leaf samples were ground in 4 ml of cold 50 mM K-phosphate buffer (pH 7.0) containing 2 mM Na–ethylenediaminetetraacetic acid (EDTA) and 1% (w/v) polyvinyl–polyvinylpyrrolidone (PVPP). The homogenates were centrifuged at 10,000 × g (4°C) for 10 min and the supernatants were transferred into new tubes. The enzyme extracts were stored at −80°C prior to activity measurements as described below. All enzyme assays were performed on extracts from three independent biological replicates.

Superoxide dismutase (SOD) activity was determined by measuring the inhibition of photochemical reduction of nitro-blue tetrazolium (NBT) in the presence of riboflavin under light according to Giannopolitis and Ries (1977) with a minor modification. The reaction mixture (3 ml) containing 50 mM K-phosphate buffer (pH 7.8), 0.1 mM EDTA (pH 8.0), 14.9 mM methionine, 63 μM NBT, 90 μl enzyme extract, and 8 μM riboflavin was incubated under 4,000 lux light intensity for 5 min. In addition, two blank samples without enzyme extract were prepared, and while blank 1 was incubated under dark for 5 min, blank 2 was incubated under the light. Photochemical reduction of NBT was quenched by placing tubes in the dark. Then, the absorbance of samples and blanks were measured at 560 nm. One unit (U) of SOD activity was defined as the amount of the enzyme causing 50% inhibition of NBT reduction.

Catalase (CAT) activity was determined by decomposition of H_2_O_2_ according to Aebi (1984). The reaction was initiated by adding 300 μl 100 mM H_2_O_2_ and 30 μl enzyme extract to 2.67 ml 50 mM phosphate buffer (pH 7.0). CAT activity was calculated by quantifying the rate of change in absorbance at 240 nm over 2 min using an extinction coefficient of 39.4 mM^−1^ cm^−1^.

For peroxidase (POD) activity, the oxidation of guaiacol in the reaction mixture was estimated by quantification of the rate of change of absorbance measured at 470 nm for 2 min according to (Maehly and Chance, 1954). The reaction mixture contained 2.84 ml K-phosphate buffer (10 mM, pH 7.0), 50 μl guaiacol (20 mM), and 90 μl enzyme extract. The reaction was initiated by addition of 20 μl H_2_O_2_ (40 mM). POD activity was calculated by using an extinction coefficient of 26.6 mM^−1^ cm^−1^.

Ascorbate peroxidase (APX) activity was measured by following the decrease in the amount of ascorbate as estimated from the rate of change in absorbance at 290 nm for 2 min (Nakano and Asada, 1981). One microliter of reaction mixture contained 50 mM K-phosphate buffer (pH 7.0), 1 mM EDTA–Na_2_, 0.5 mM ascorbic acid, 0.1 mM H_2_O_2_, and 25 μl enzyme extract. The activity of ascorbate peroxidase was calculated using an extinction coefficient of 2.8 mM^−1^ cm^−1^.

### Metabolite Profiling by Gas Chromatography Mass Spectrometry

Fully expanded leaves were harvested from the fourth node of three independent replicate plants and immediately frozen in liquid nitrogen. Samples were stored at −80°C prior to lyophilization, extraction, and analysis. Samples were extracted as polar and non-polar phases, derivatized, and analyzed by gas chromatography mass spectrometry (GC-MS) as previously described (Dobson et al., 2008; Trapero-Mozos et al., 2018b). Metabolite profiles were acquired using a GC–MS (DSQII Thermo-Finnigan, UK) system carried on a DB5-MSTM column (15 m × 0.25 mm × 0.25 µm; J&W, Folsom, CA, USA) as described by Foito et al. (2013).

### Microarray Methods

Fully expanded leaves were harvested from the fourth node of three independent replicate plants and immediately frozen in liquid nitrogen. Samples were stored at −80°C prior to extraction and analysis. RNA was extracted from potato leaves as described previously (Ducreux et al., 2008). Microarray analysis was performed using a custom Agilent microarray designed to the predicted transcripts from assembly version 3.4 of the DM potato genome (The Potato Genome Consortium, 2011) as described by Morris et al. (2014). The experimental design and complete datasets are available at ArrayExpress (http://www.ebi.ac.uk/arrayexpress/; accession number E-MTAB-8298). Briefly, a single-channel microarray design was utilized with leaf RNA samples all labeled with Cy3 dye. RNA labeling and downstream microarray processing was performed as recommended using the Low Input Quick Amp Labeling Kit (v 6.5; Agilent). Following microarray scanning using an Agilent G2505B Scanner, data were extracted from images using Feature Extraction (FE (v. 10.7.3.1) software and aligned with the appropriate array grid template file (033033_D_F_20110315). Intensity data and QC metrics were extracted using the recommended FE protocol (GE1_107_Sep09). Entire FE datasets for each array were loaded into GeneSpring (v. 7.3; Agilent) software for further analysis. Data were normalized using default single-channel settings: intensity values were set to a minimum of 0.01, data from each array were normalized to the 50th percentile of all measurements on the array, and the signal from each probe was subsequently normalized to the median of its value across all samples. Unreliable data flagged as absent in all replicate samples by the FE software were discarded. Statistical filtering of data was performed using two-way analysis of variance (ANOVA; P ≤0.05) for the factors “cultivar” and “treatment,” with Bonferroni multiple testing correction.

### Statistical Methods

Statistical analysis was carried out on the metabolite data sets collated from GC/MS polar and non-polar fractions. Firstly, principal components analysis (PCA), using the sample correlation matrix which gives equal weight to all metabolites, was used to summarize broad scale variation among the samples. A second approach involved an ANOVA of each individual metabolite using the main factors variety, treatment (C, D, H, H+D), or an interaction of the two factors. Gene expression data were also analyzed by ANOVA, in this case the data used compared the varieties, the control samples and the heat and drought treatments, and the interaction between the two. Genes were selected according to their p-values from the ANOVA F-test, using a significance threshold based on the estimated false discovery rate (FDR) of less than 1% (p values 0.019%) (Benjamini and Yekutieli, 2001). Statistical analyses were performed using GenStat for Windows, 18th Edition (VSN International Ltd., Hemel Hempstead, UK).

## Results

The key objective of the present work was to identify the physiological, biochemical, and molecular responses to single and combined abiotic stresses that underpinned the tolerance phenotype in potato. We therefore chose to compare the responses of stress tolerant and stress susceptible genotypes. Desiree and Unica were chosen as tolerant to heat and drought, and Agria and Russet Burbank were selected as heat and drought susceptible genotypes, based on previous literature (Basu and Minhas, 1991; Ahn et al., 2004; Gutiérrez-Rosales et al., 2007; Stark et al., 2013; Rolando et al., 2015; Demirel et al., 2017). However, previous reports have not assessed responses to combined abiotic stress and hence a second objective was to determine the resistance of genotypes to combined heat and drought.

A key determinant of potato yield under abiotic stress is the capacity to maintain photoassimilation and carbohydrate transport to developing tubers (van Loon, 1981; Reynolds et al., 1990; Wolf et al., 1991; Hancock et al., 2014). Therefore, in initial experiments, we chose to undertake a detailed time course of gas exchange parameters to define conditions under which genotypes would experience a moderate to severe stress. This time point was then used for further biochemical, metabolomics, and transcriptional analysis in an attempt to identify genes and processes associated with abiotic stress tolerance. By contrasting two tolerant and two susceptible genotypes, the intention was to identify broadly adopted strategies associated with stress resistance allowing us to identify potential targets for genetic improvement of potato genotypes suitable for cultivation in adverse growing environments.

### Morphological Responses of Tolerant and Sensitive Potato Genotypes to Abiotic Stress

**Figure 1:**
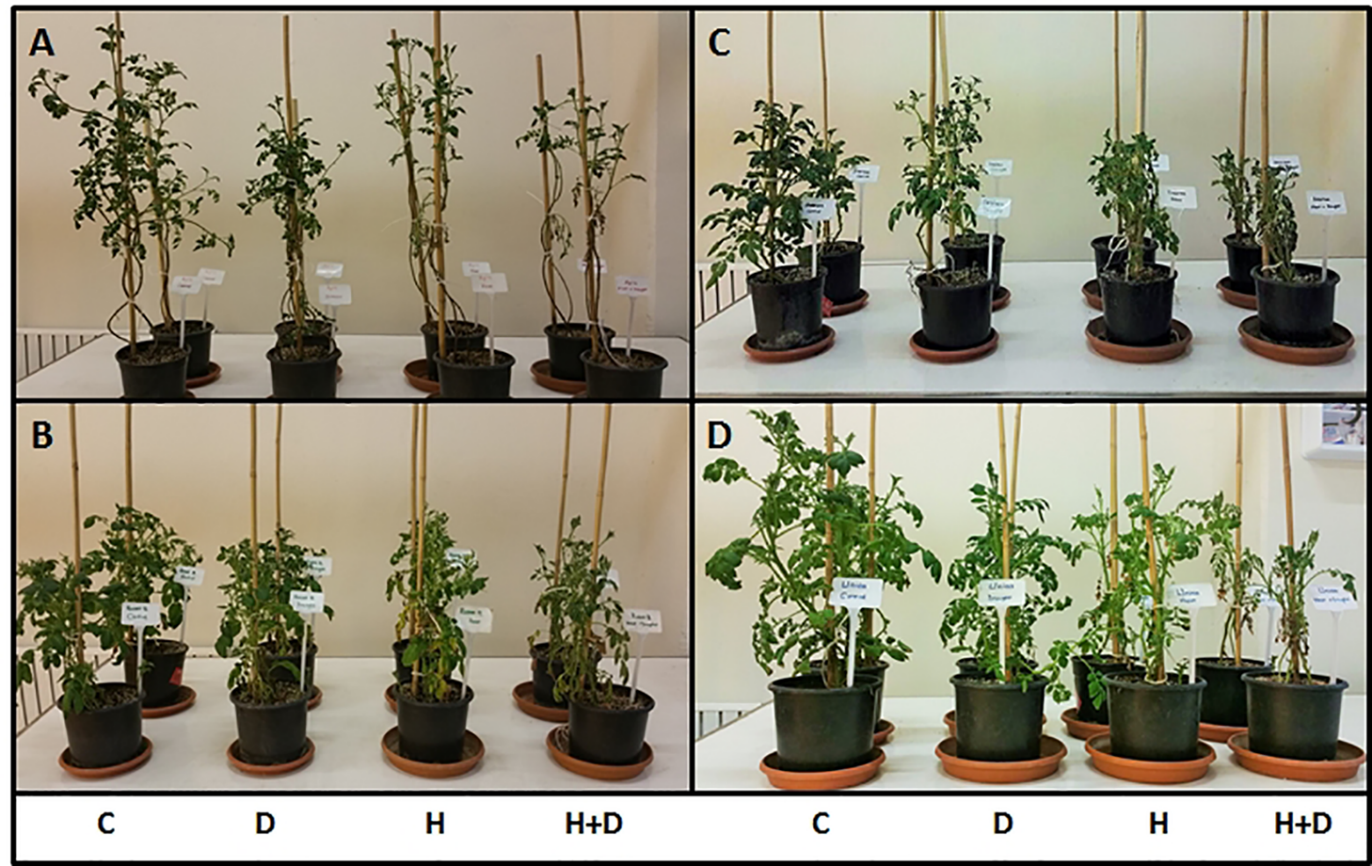
Above ground phenotype of two drought and heat sensitive **(A**, **B)** and two drought and heat tolerant **(C**, **D)** potato varieties after 12 days of abiotic stress treatment. A, Agria; B, Russet Burbank; C, Desiree; D, Unica. Treatments were control (C), drought (D), heat (H), and heat with drought (H+D).

**Figure 1** shows representative images of the four potato genotypes used in this study following 12 days exposure to abiotic stress treatments. Stress treatments affected above ground morphology and biomass although the impact varied by plant genotype and treatment. Combined heat and drought treatments had the strongest impact where stems were shorter and leaves less abundant than in respective control plants. In the heat and drought sensitive cultivar Agria (**Figure 1A**) drought resulted in reduced stem length while heat extensively reduced leaf biomass. Similar although less severe symptoms were observed for the tolerant cultivar Unica (**Figure 1D**). In another stress sensitive genotype, Russet Burbank (**Figure 1B**) above ground biomass was reduced by both drought and heat treatments while tolerant Desiree plants had reduced foliage under drought and shorter stems following heat stress (**Figure 1C**).

### Physiological Responses of Tolerant and Sensitive Potato Genotypes to Abiotic Stress

In order to define an appropriate time point for subsequent analysis of physiological, metabolic, and transcriptomic responses to single and combined stresses, stress severity was monitored by measuring gas exchange parameters in the four different cultivars at different time points following the imposition of stress treatments. The intention was to identify a time point at which all cultivars were experiencing stress so that differences in stress-induced responses between stress tolerant and stress sensitive cultivars could be identified in subsequent analyses.

**Figure 2:**
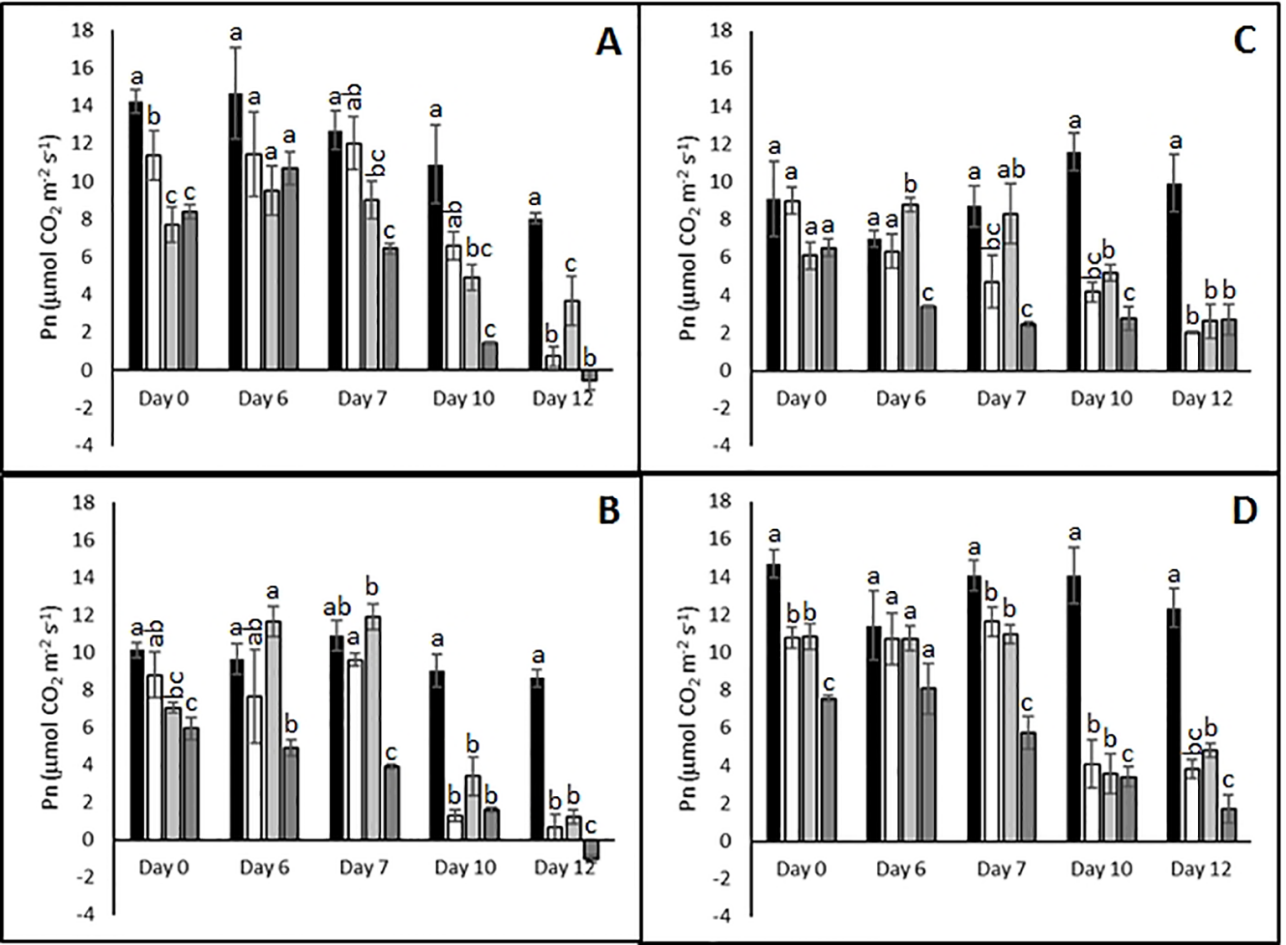
Influence of abiotic stress on photosynthetic capacity of potato cultivars with contrasting stress sensitivity. Plants were subjected to moderate temperatures and daily watering (control, ■) or drought (□), heat (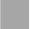), or combined drought and heat (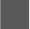) stress for up to 12 days as described. Net photosynthetic rate was quantified using a LiCor LI-6400XT portable photosynthesis system under saturating light conditions on the fourth fully expanded leaf from three independent replicate plants of the sensitive cultivars Agria **(A)** and Russet Burbank **(B)**, and the resistant cultivars Desiree **(C)**, and Agria **(D)**. Data are presented as mean ± SE of three independent biological replicates. Significant differences within a time point as estimated using one-way ANOVA with Tukey's protected least significant difference (LSD) test are indicated by different letters (P < 0.05).

Abiotic stress treatments resulted in reduced photosynthetic rates in all cultivars toward the end of the treatments (**Figure 2**). For example, after 10 days Pn was strongly reduced in all cultivars irrespective of the stress treatment or plant genotype. By day 12 of the combined heat and drought treatment the sensitive genotypes Agria and Russet Burbank (**Figures 2A, B**) exhibited net respiration while the resistant genotypes continued to fix carbon albeit much less efficiently than control plants (**Figures 2C, D**). At earlier times in the stress treatment, there was little correlation between reported stress sensitivity and photosynthetic carbon assimilation. For example on day 6 the sensitive genotype Agria (**Figure 2A**) and the resistant Unica (**Figure 2D**) failed to exhibit significant changes in CO_2_ assimilation between plants subjected to combined stress and control plants while sensitive Russet Burbank (**Figure 2B**) and resistant Desiree (**Figure 2C**) did show significant impairment following combined stress. We therefore chose to focus subsequent investigations on the plants that had been subjected to 12 days of stress treatment.

**Figure 3:**
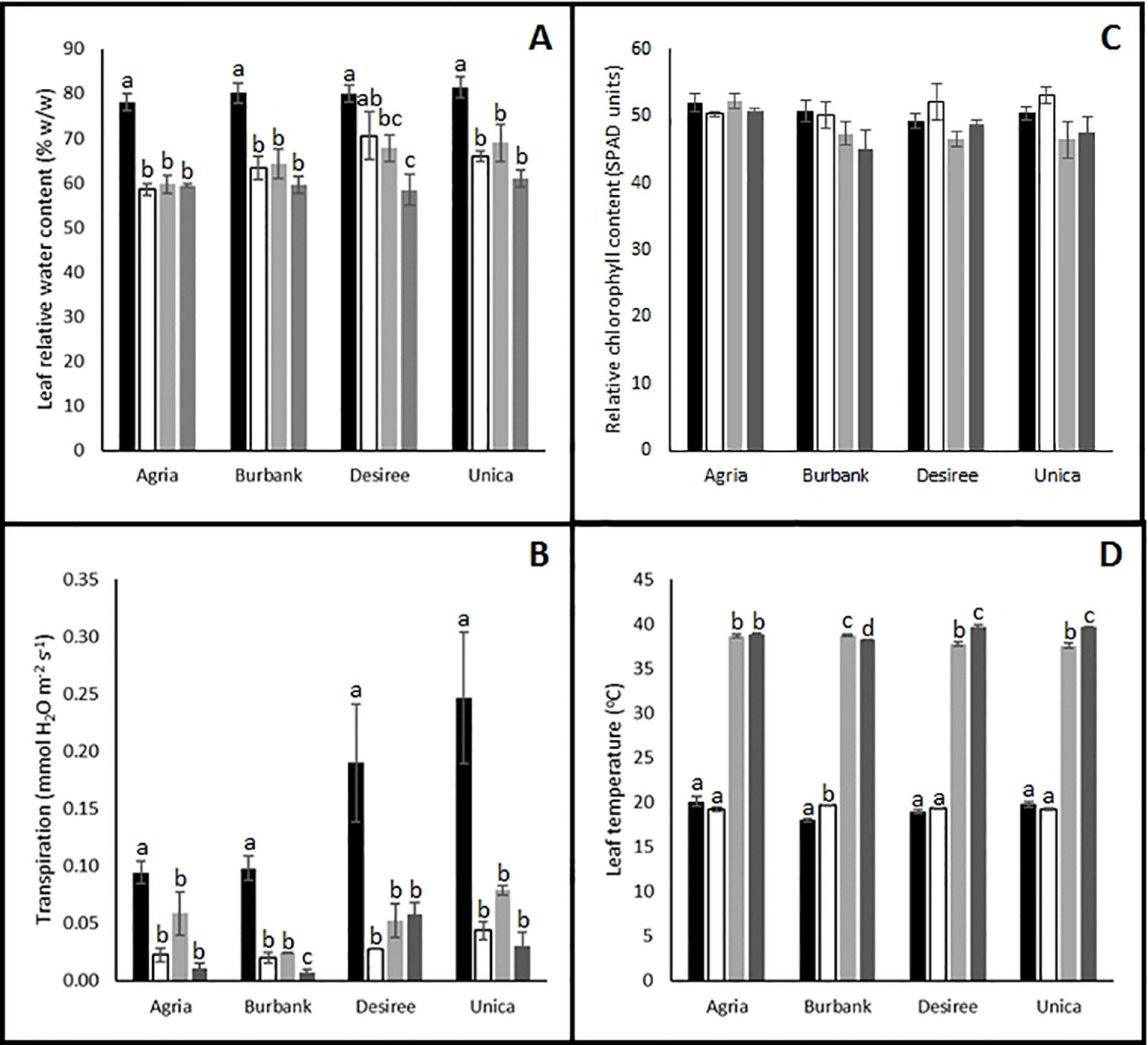
Influence of abiotic stress on leaf physiological traits in potato cultivars with contrasting stress sensitivity. Plants were maintained under control conditions (■) or subjected to 12 days drought (□), heat (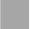), or combined drought and heat (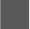) stress. Leaf relative water content **(A)**, transpiration rate **(B)**, chlorophyll content **(C)**, and temperature **(D)** were quantified as described in the text. Data are presented as mean ± SE of three independent biological replicates. Significant differences within a cultivar as estimated using one-way ANOVA with Tukey's protected least significant difference (LSD) test are indicated by different letters. Letters are absent where the level of significance was below 0.05.

To further investigate the causes of reduced photosynthetic performance, several physiological measurements were recorded following 12 days of stress imposition. Irrespective of the stress imposed (drought, heat or combinatorial stress), leaf relative water content was significantly reduced compared with control plants (**Figure 3A**). These data reflected the significantly reduced rates of transpiration observed following stress treatment (**Figure 3B**). Leaf chlorophyll content (**Figure 3C**) was not significantly reduced following abiotic stress treatment suggesting that the stress was not sufficient to induce leaf senescence after 12 days providing further confidence that the 12 days time point was appropriate for examination of differences in stress-induced mitigation strategies between tolerant and resistant cultivars. Interestingly, the resistant genotypes Unica and Desiree had a higher leaf temperature in combined heat and drought treatment compared with heat alone which was not observed in the sensitive genotypes Russet Burbank and Agria (**Figure 3D**). These data are consistent with the induction of non-photochemical quenching leading to absorbed light energy being dissipated as heat in the stress resistant cultivars thereby protecting photosynthetic electron transport chains from photodamage.

Taken together the data indicates that all varieties are impaired by drought, heat, or their combination. However, tolerant varieties are clearly able to maintain photosynthesis for longer under severe stress conditions. This is accompanied by a stronger capacity for the induction of protective mechanisms such as non-photochemical quenching.

### Biochemical Responses of Tolerant and Sensitive Potato Genotypes to Abiotic Stress

**Figure 4:**
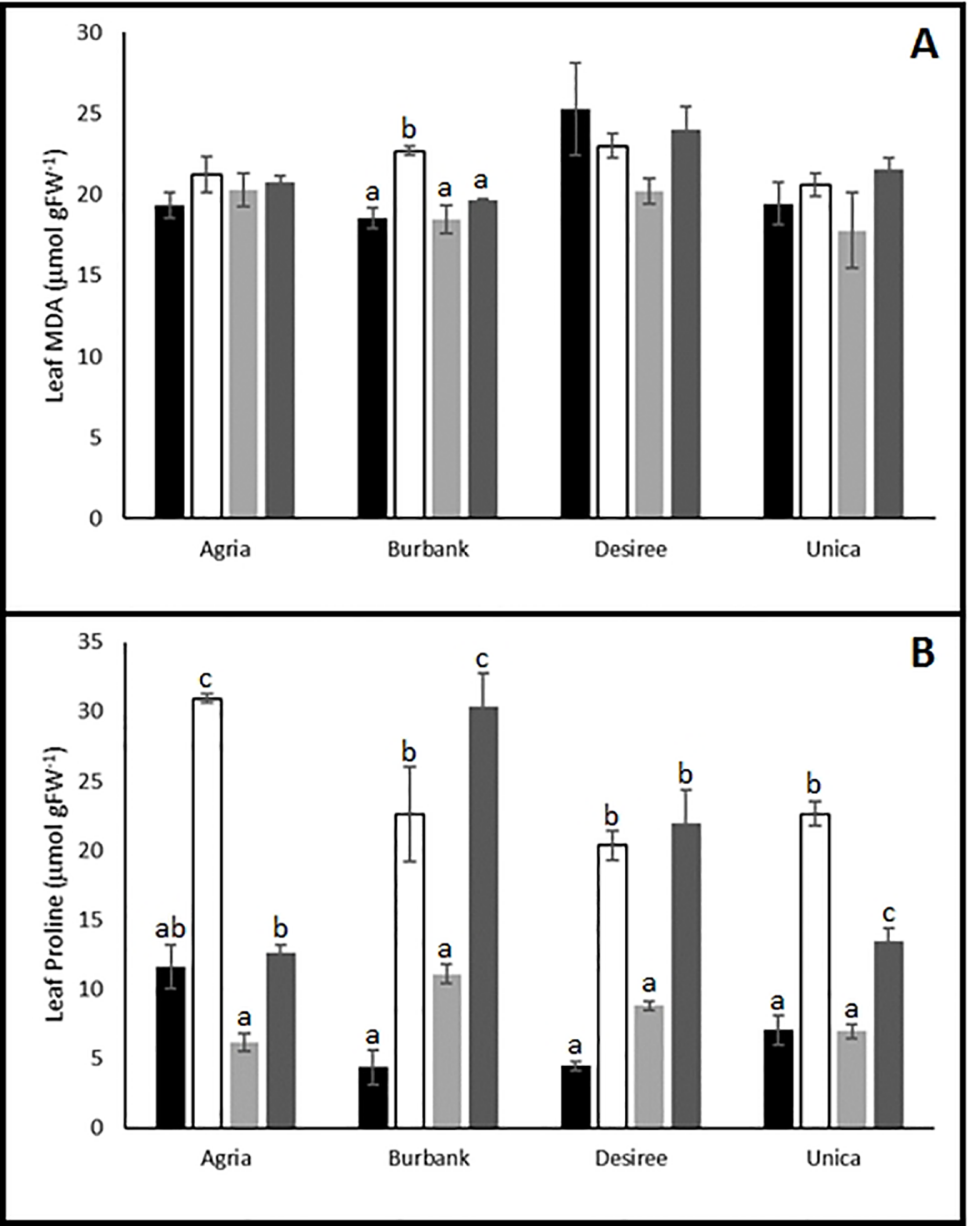
Influence of abiotic stress on leaf biochemical traits in potato cultivars with contrasting stress sensitivity. Plants were maintained under control conditions (■) or subjected to 12 days drought (□), heat (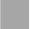), or combined drought and heat (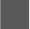) stress. Leaf malondialdehyde (MDA) **(A)** and proline **(B)** content were estimated as described in the text in three independent biological replicates. Data are presented as mean ± SE of three independent biological replicates. Significant differences within a cultivar as estimated using one-way ANOVA with Tukey's protected least significant difference (LSD) test are indicated by different letters. Letters are absent where the level of significance was below 0.05.

Biochemical profiling of leaves following stress treatments also indicated differences between genotypes in their capacity to induce protective mechanisms. MDA is an oxidation product of unsaturated lipids that accumulates in response to oxidative stress (Yalcinkaya et al., 2019) and has previously been shown to accumulate in potato plants exposed to drought stress (Zhang et al., 2017; Nie et al., 2018). We therefore examined the impact of abiotic stress on MDA accumulation in resistant and sensitive potato genotypes. The resistant genotypes Desiree and Unica exhibited similar leaf MDA content following stress treatments compared with control leaves. In the susceptible variety Agria there was a trend toward higher leaf MDA content following stress while in Russet Burbank there was a significant increase in leaf MDA following drought stress (**Figure 4A**).

Proline is a well-established compatible osmolyte that accumulates in plants in response to a range of abiotic stresses (Kaur and Asthir, 2015), most notably drought and salt stress (Per et al., 2017). We therefore examined the accumulation of this osmolyte in potato leaves as a marker of abiotic stress induction. Irrespective of whether varieties were classed as stress sensitive or tolerant, leaves accumulated proline following drought or drought and heat treatment. A notable exception was the cultivar Agria which accumulated proline when subjected to drought stress but exhibited a proline content in drought and heat stressed leaves similar to that in control leaves (**Figure 4B**) indicating that a key drought protective mechanism was inactivated by the imposition of the combined stress. Heat stress alone did not lead to the accumulation of proline in any of the cultivars.

**Figure 5:**
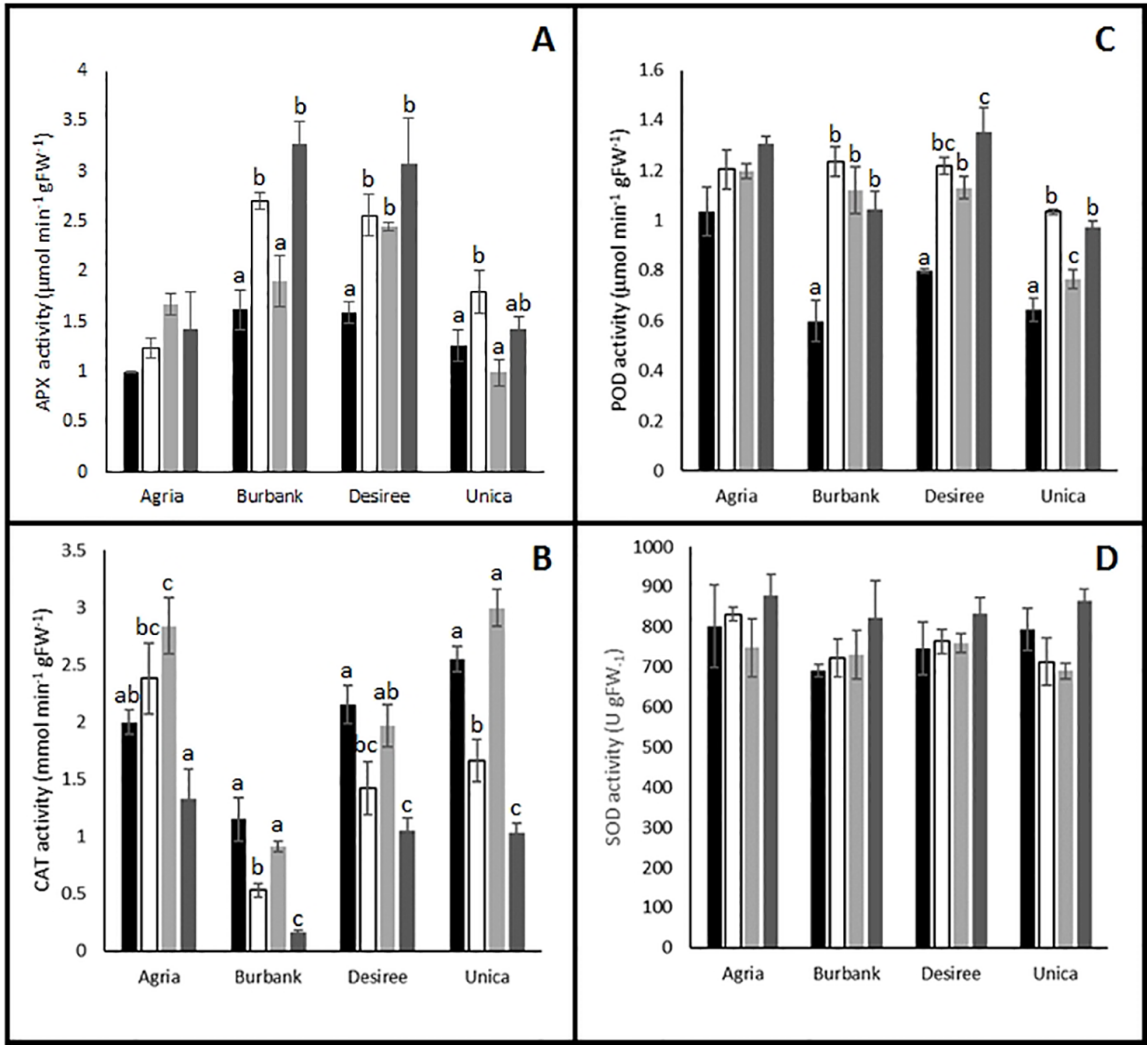
Influence of abiotic stress on leaf antioxidant enzyme activity in potato cultivars with contrasting stress sensitivity. Plants were maintained under control conditions (■) or subjected to 12 days drought (□), heat (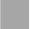), or combined drought and heat (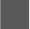) stress. Leaf ascorbate peroxidase **(A)**, catalase **(B)**, peroxidase **(C)**, and superoxide dismutase **(D)** activity were estimated as described in the text in three independent biological replicates. Data are presented as mean ± SE of three independent biological replicates. Significant differences within a cultivar as estimated using one-way ANOVA with Tukey's protected least significant difference (LSD) test are indicated by different letters. Letters are absent where the level of significance was below 0.05.

To test the hypothesis that plants responded to potential increases in oxidative stress by altering antioxidant metabolism, activities of key antioxidant enzymes were quantified and several changes in activity were observed following the imposition of stress. Antioxidant enzyme activities of the stress sensitive genotype Agria were relatively unaffected by stress treatment. Indeed, the only significant difference from control treatments were found in catalase (CAT) activity following heat stress (**Figure 5B**) while activities of ascorbate peroxidase (APX, **Figure 5A**), peroxidase (POD, **Figure 5C**), and superoxide dismutase (SOD, **Figure 5D**) were unaffected by stress treatments. The other stress sensitive cultivar Russet Burbank exhibited no change in CAT activity following heat stress and a significant inhibition of activity following drought either alone or in combination with high temperature (**Figure 5B**). Similar patterns of catalase activity were observed in the stress resistant cultivars Desiree and Unica (**Figure 5B**). On the contrary, APX activity was significantly increased by the drought treatments in Russett Burbank, Desiree, and Unica and in the case of Russet Burbank and Desiree this enhancement was also observed in combined heat and drought treatments (**Figure 5A**). These data suggest that in the cultivars Russet Burbank, Desiree, and Unica, H_2_O_2_ is primarily processed by the activity of ascorbate peroxidase while in Agria, H_2_O_2_ is primarily processed *via* the activity of catalase. Peroxidase activity was enhanced by all stress treatments in all genotypes with the exception of Agria for which no changes were observed (**Figure 5C**). However, SOD activity was unresponsive to stress in all genotypes (**Figure 5D**).

Despite the obvious differences in leaf physiological responses to abiotic stress between genotypes, there were no clear correlations with the biochemical responses that were measured. A clear response to abiotic stress in the tolerant genotypes was the induction of APX and peroxidase activity, however this response was also shared by one of the sensitive cultivars, Russet Burbank. Taken together, the data suggest that all genotypes experience an increased oxidative load in response to abiotic stress and that they all mount some kind of oxidative defense as indicated by the induction of antioxidant enzymes and with the exception of Russet Burbank under drought stress a lack of accumulation of oxidative markers. These data perhaps suggest that plants have induced acclimatory responses the abiotic environment that minimize ROS production thereby protecting photosynthetic structures to allow continued CO_2_ assimilation following a return to more benign environmental conditions. To further elucidate stress/acclimation responses, future studies should examine the detailed temporal dynamics of antioxidant defense responses across the entire period of stress induction and acclimation.

### Transcript Profiles of Potato Leaves Subjected to Single and Combined Abiotic Stress

Our phenotypic analysis clearly revealed abiotic stress induced responses in all cultivars following 12 days of treatment. Furthermore, at this time point a clear difference in the capacity to maintain CO_2_ assimilation was observed between stress tolerant and stress sensitive genotypes. We therefore chose the 12 day time point to analyze leaf transcript profiles of all genotypes based upon the hypothesis that at this time point different stress response pathways leading to either tolerance in the case of Desiree and Unica or susceptibility in the case of Agria and Russet Burbank would be activated. Thus in Desiree and Unica gene expression might be associated with acclimation while acclimatory gene expression may be less effective in Agria and Russet Burbank.

RNA was extracted from leaves and relative transcript abundance was determined by microarray analysis as described in materials and methods. Fluorescence data were subjected to two-way ANOVA using the factors cultivar and stress treatment to identify transcripts that were specifically expressed as a function of genotype or as a function of stress treatment. As ANOVA considers the dataset as a whole, we hypothesized that the former gene list would be dependent on the genetic background independent of the stress treatment while the latter gene list would be stress dependent independent of the genetic background. This means that the stress dependent gene list would describe the stress responses that were common to all genotypes. Using a significance value of 0.05 and Benjamini-Hochberg multiple testing correction, we identified more than 19,000 transcripts that were significantly differentially abundant dependent on genotype and more than 18,000 transcripts whose abundance was dependent on treatment (**Supplementary Table S1**).

To determine how our stress related transcripts compared with previous studies we selected a number of transcripts previously identified as stress responsive to determine how they behaved in our experiment. Drought responsive transcripts were selected based upon previous work which identified a set of 23 highly significant drought responsive transcripts in potato leaves subjected to up to 10 days of drought stress (Pieczynski et al., 2018). This set of transcripts therefore represented genes that might be involved in stress acclimation. Eighteen of the 23 transcripts identified were significantly differentially expressed in our experiment as determined using ANOVA as described above. Thirteen of these transcripts had previously been reported as being induced by drought (Pieczynski et al., 2018) of which twelve were weakly induced by drought in Desiree, Unica, and Agria but more strongly induced in Russet Burbank (**Supplementary Figure S2**). The exception was a transcript encoding a lipid transfer protein (DMT400014686). All of these transcripts were strongly induced when plants were subjected to combined heat and drought suggesting synergistic interactions between these two stresses and potentially a stronger acclimatory response when the stresses were combined. On the contrary, a ubiquitin-protein ligase (DMT400060674) previously reported as drought inducible was not induced by any of the stress treatments in the present experiment. Five transcripts previously reported to decrease in abundance following drought stress exhibited varying patterns of expression in the present experiment. For example, a transcript encoding a calcium binding protein (DMT400019204) exhibited weakly reduced abundance following drought in all genotypes whereas a transcript encoding an adenyl cyclase-associated protein (DMT400001911) exhibited decreased abundance in stress tolerant but increased abundance in stress susceptible cultivars (**Supplementary Figure S2**).

Heat stress marker transcripts were selected based on those encoding functionally relevant potato proteins as determined using a yeast bioassay (Gangadhar et al., 2014). Transcripts had been isolated from potato leaf expression libraries following 2 or 48 h of heat stress. Given that the 2 h time point was more likely to represent short term heat shock, we selected only transcripts isolated from the 48 h library. Out of the 65 transcripts previously identified, 45 came through our ANOVA analysis. The majority of transcripts exhibited increased abundance following heat or combined heat and drought stress in all genotypes (**Supplementary Figure S3**). Key exceptions included a series of transcripts encoding proteins associated with photosynthetic functions such as DMT400021392 encoding a chlorophyll binding protein and DMT400054480 encoding PSI subunit III. These data indicate that many transcripts upregulated at 48 h maintain a high level of expression following prolonged abiotic stress and that their expression may need to be maintained to allow plants to acclimate to the abiotic environmental conditions.

Taken together, the comparative analysis indicates that our dataset exhibited similarities to previous studies where potato plants were exposed to long term abiotic stress conditions. A key question was whether the patterns of gene expression resulted in acclimatory responses that allow plants to survive under periods of extended suboptimal abiotic environments. We therefore focused attention on the set of 461 transcripts that exhibited a genotype x stress treatment interaction indicating that they responded differentially to stress in different genotypes. We hypothesized that this group of transcripts would highlight specific cultivar responses to stress resulting in specific changes that imparted either stress tolerance (acclimation) or stress sensitivity (inability to acclimate).

Classification of the 461 transcripts that showed a genotype x stress interaction using the MapMan tool (Thimm et al., 2004) revealed relatively high numbers of transcripts associated with protein metabolism (MapMan bin 29) of which more than 20 transcripts were specifically associated with protein degradation (bin 29.5, **Supplementary Table S2**). Included in this group was a transcript (DMT400022219) encoding a cysteine-type endopeptidase with homology to *Arabidopsis* metacaspases and seven transcripts encoding subtilases. Both metacaspases and subtilases play a role in programmed cell death in plants (Kabbage et al., 2017). More than 20 transcripts were classified as functioning in transport (bin 34) of which more than half were associated with the transport of sugars, amino acids, or peptides (**Supplementary Table S2**). These data indicate significant metabolic turnover and redistribution in response to abiotic stress in potato. Similarly, 23 transcripts associated with stress (bin 20) were represented of which six were classified as being associated with abiotic stress (bin 20.2). Two of these transcripts (DMT400000185, DMT400000187) encoded proteins with homology to an *Arabidopsis* transcript (At2g46240) encoding a Bcl-2-associated athanogene protein known to be responsive to heat stress (Echevarría-Zomeño et al., 2016) and involved in the control of cell death (Pan et al., 2016). A large number of transcripts associated with signaling processes including those encoding proteins associated with hormone metabolism (bin 17), signaling (bin 30) and transcription (bin 27.3) were also highly represented (**Supplementary Table S2**). Interestingly, the hormone associated transcripts did not include any transcripts associated with ABA, the primary hormone associated with drought stress although these transcripts were well represented in the treatment responsive transcripts (**Supplementary Table S1**). On the contrary there were several transcripts in the genotype x treatment list that were associated with auxins, cytokinins, ethylene, jasmonate and salicylate (**Supplementary Table S2**) which have been previously described as fine tuning the drought response (Ullah et al., 2018). Among the transcription factors two transcripts (DMT400026836, DMT400063666) had similarity to an *Arabidopsis* transcript (At2g20880) encoding an AP2 domain transcription factor that has been shown to be induced by drought or heat stress (Hsieh et al., 2013). This transcription factor was induced by either drought or heat and was strongly induced in all varieties by combined drought and heat stress (**Supplementary Table S2**) providing confirmation of the physiological measurements.

**Figure 6:**
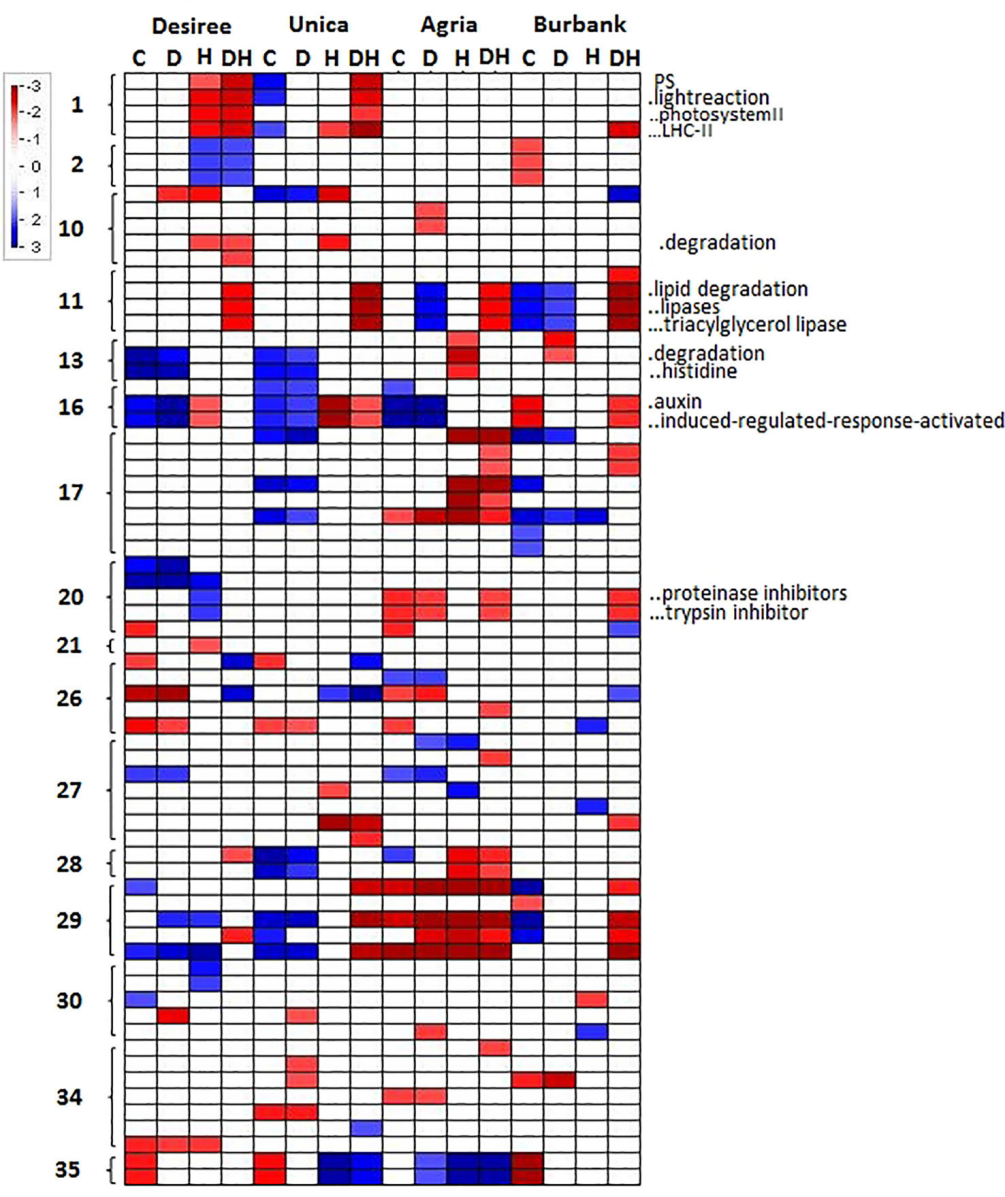
PageMan representation of significantly up or downregulated transcript ontologies in the subset of transcripts whose abundance exhibits significant variation in a genotype x treatment dependent manner. Transcripts were grouped according to their MapMan bin and transcript groups that exhibited a significant average change in abundance are indicated according to a color log_2_ scale as indicated. Absence of color indicates that average change in abundance for a specific MapMan bin were not significant for any given sample. Columns labels indicate genotype and treatment (C, control; D, drought; H, heat; DH, drought and heat). Row labels indicate the primary MapMan bin (1, photosynthesis; 2, major CHO metabolism; 10, cell wall; 11, lipid metabolism; 13, amino acid metabolism; 16, secondary metabolism; 17, hormone metabolism; 20, stress; 21, redox; 26, miscellaneous; 27, RNA; 28, DNA; 29, protein; 30, signaling; 34, transport; 35, not assigned). Shortened bin names of selected categories are indicated to the right of the figures.

To further characterize genotypic differences in response to abiotic stress, the PageMan tool (Usadel et al., 2006) was used to functionally characterize cultivar specific changes in gene expression. A Wilcoxon test was applied to determine significant differences in the median fold change in abundance of transcripts within a particular ontological group relative to the median fold change across all ontological groups. This function therefore describes whether a particular transcript class is on average more or less abundant following the imposition of a particular treatment. Several transcript classes were significantly induced or repressed in certain genotypes under different abiotic stresses. In order to determine molecular functions that were consistently associated with stress susceptibility or tolerance, we focused on gene classes that were commonly up- or down-regulated in both stress resistant (Desiree, Unica) or both stress susceptible (Agria, Russett Burbank) cultivars. Desiree and Unica both exhibited a significant reduction in the abundance of transcripts associated with PSII, particularly transcripts encoding light harvesting chlorophyll-protein complex components, when exposed to drought and heat and no such reduction was observed in Agria or Russett Burbank. Similarly, a strong reduction in these transcripts was observed under conditions of high temperature alone in Desiree (**Figure 6**). Other metabolic adjustments that were specific to abiotic stress tolerant genotypes included a reduction in transcripts associated with cell wall degradation and an increase in transcripts associated with amino acid degradation (**Figure 6**). In contrast the sensitive cultivars exhibited a common reduction in the abundance of transcripts encoding proteinase inhibitors. These results suggest that tightly controlled amino acid and protein turnover is important in potato response to abiotic stress as has previously been suggested from analysis of multiple abiotic stress transcript datasets (Kilian et al., 2012). Stress susceptible cultivars also exhibited a common increase in the abundance of transcripts associated with lipid degradation and a reduction in auxin associated transcripts (**Figure 6**).

**Figure 7:**
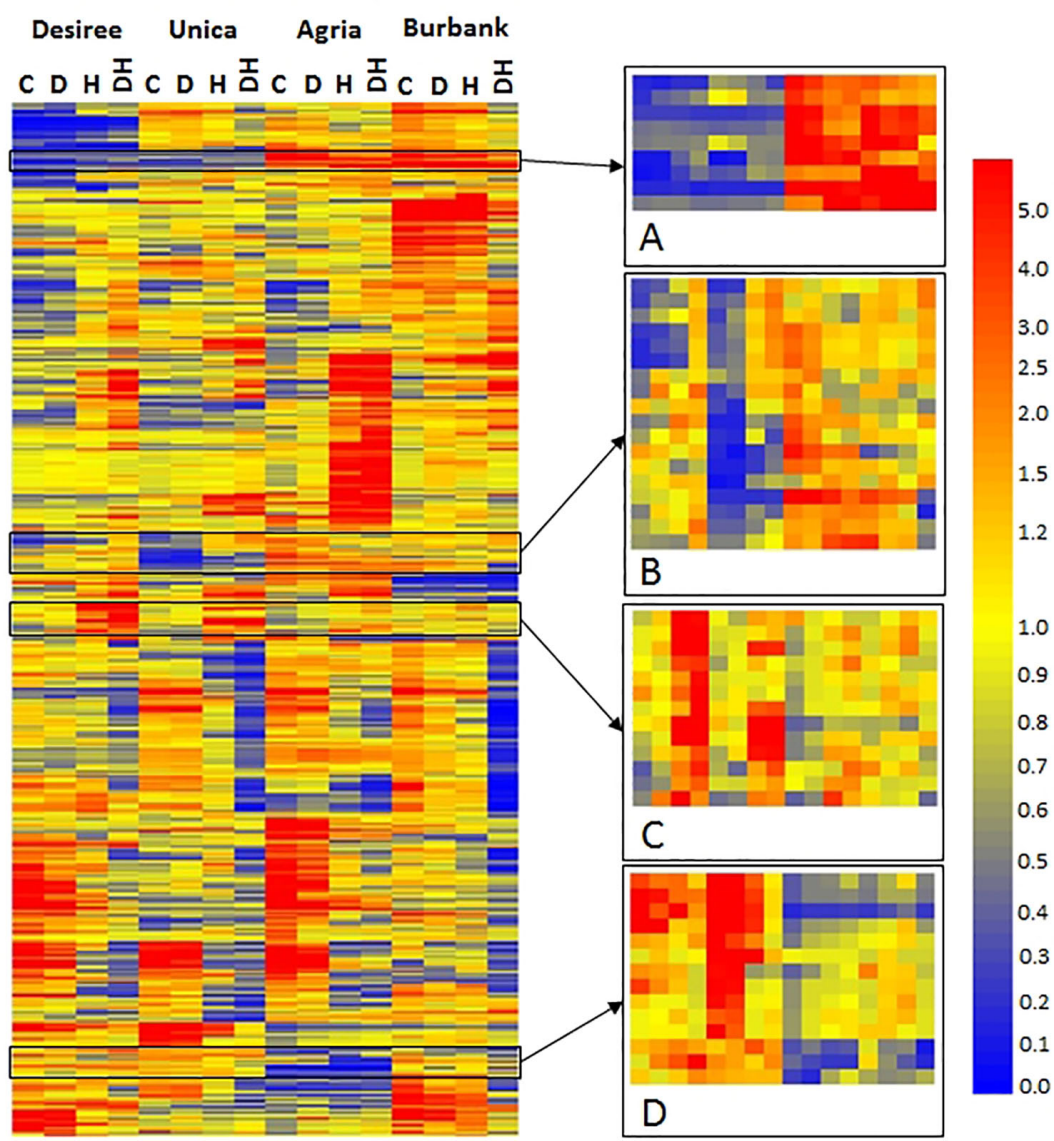
Heatmap of differentially expressed transcripts exhibiting a genotype x treatment interactive effect on abundance. Columns labels indicate genotype and treatment (C, control; D, drought; H, heat; DH, drought and heat). Transcripts were clustered using GeneSpring and mean relative abundance of three independent biological replicates is indicated according to the scale bar shown. Selected regions exhibiting common patterns of expression in stress resistant (Desiree, Unica) or stress tolerant (Agria, Russet Burbank) cultivars are highlighted **(A**–**D)**. Gene order in these regions is listed in **Table 1**.

To identify specific transcripts associated with stress tolerance or stress susceptibility, hierarchical cluster analysis was performed to highlight transcripts commonly expressed in either stress tolerant or stress susceptible genotypes. A diverse array of patterns of gene expression was observed, however a set of 54 genes were identified that exhibited common expression profiles among abiotic stress tolerant or susceptible cultivars (**Figure 7**). Our rationale was that despite the wide genotypic differences between the varieties, transcripts exhibiting common patterns of expression in stress tolerant or stress susceptible cultivars would also be associated with stress tolerance/susceptibility in a wider pool of potato germplasm and hence are the most significant genes upon which to focus in terms of breeding. Several transcripts exhibited low abundance in abiotic stress tolerant genotypes and high abundance in stress sensitive genotypes irrespective of treatment (**Figure 7A**). The majority of these transcripts were of unknown function however, this group also included a transcript associated with biotic stress (DMT400019055), a transcript encoding a putative auxin efflux carrier (DMT400048071), and a transcript encoding a protease inhibitor (DMT400026258) (**Table 1**). Several transcripts exhibited relatively low abundance in stress tolerant lines under control and drought conditions that were more highly expressed under the same conditions in stress sensitive genotypes (**Figure 7B**). This group included several transcripts encoding putative cytochrome P450s of unknown function, two glycosyltransferases, and three transcripts encoding proteins of unknown function (**Table 1**). This group also contained a transcript encoding a caspase (DMT400032693) with homology to *Arabidopsis* metacaspase 3 (At5g64240), a type I metacaspase. Type I metacaspases have been implicated in the control of programmed cell death in *Arabidopsis* (Coll et al., 2010). Interestingly, a transcript (DMT400036544) with homology to an *Arabidopsis* transcript encoding ABI5 binding protein 2 (AFP2, At1g13740) with a role in response to water deprivation was also present among these transcripts. A further group of transcripts were highly expressed in response to heat or heat and drought in stress tolerant genotypes but less so in stress susceptible genotypes (**Figure 7C**). Several of these transcripts encoded proteins involved in primary metabolism such as cell wall invertase (DMT400023092), a tryptophan synthase (DMT400029363), and enzymes involved in lipid biosynthesis (DMT400033492) and C1-metabolism (DMT400019729) (**Table 1**). This group of transcripts also encoded transcription factors (DMT400035119, DMT400049446) with *Arabidopsis* homologues involved in plant growth, development, and stress responses including a nuclear factor Y transcription factor (Zhao et al., 2017) and a DELLA transcription factor required for gibberellin signaling in response to environmental signals (Alvey and Harberd, 2005). Interestingly, this group also contained a transcript encoding an ubiquitin protein ligase (DMT400075387) with homology to an *Arabidopsis* gene (At4g12570) that acts as a negative regulator of leaf senescence (Miao and Zentgraf, 2010).

**Table 1:** Transcripts exhibiting common expression patterns in stress tolerant or stress susceptible genotypes. Transcripts were identified by hierarchical cluster analysis and selected as indicated in **Figure 7**.

Transcript ID	Description	MapMan bin	Group
44031	Disease resistance protein	35.2, not assigned	A
48071	Auxin efflux carrier	34.99, transport.misc	A
89679	Unknown function	35.2, not assigned	A
86745	Unknown function	35.2, not assigned	A
19055	Disease resistance protein (NBS-LRR class)	20.1, stress.biotic	A
78352	Unknown function	35.2, not assigned	A
46074	Unknown function	35.2, not assigned	A
26258	Protease inhibitor	35.2, not assigned	A
02664	Unknown function	35.2, not assigned	A
65520	Syntaxin	31.4, cell.vesicle transport	B
25271	Cytochrome P450	26.1. misc. cytochrome P450	B
25272	Cytochrome P450	26.1. misc. cytochrome P450	B
34407	Cytochrome P450	26.1. misc. cytochrome P450	B
34406	Cytochrome P450	26.1. misc. cytochrome P450	B
25264	Cytochrome P450	26.1. misc. cytochrome P450	B
96242	Unknown function	35.2, not assigned	B
33796	Glycosyltransferase	26.2, misc.UDP glycosyltransferase	B
36544	Ninja family protein AFP2	35.2, not assigned	B
71935	RNA splicing factor	27.1.1, RNA.processing.splicing	B
32693	Caspase	29.5, protein.degradation	B
39912	Glycosyltransferase	26.2, misc.UDP glycosyltransferase	B
62932	Unknown function	35.2, not assigned	B
46014	Unknown function	35.2, not assigned	B
54388	FAD-binding domain containing protein	28.6, misc.nitrilases	B
27464	Sesquiterpene synthase	16.1.5, secondary.metabolism.isoprenoids.terpenoids	B
75714	Zeatin O-xylosyltransferase	26.2, misc.UDP glycosyltransferase	B
45239	Caffeic acid O-methyltransferase	26.6, misc.O-methyltransferase	B
23092	Cell-wall invertase	2.2.1.3.2, major CHO metabolism.degradation.sucrose.invertase.cell wall	C
35119	Nuclear factor Y transcription factor	27.3.15, RNA.regulation of transcription.CCAAT box binding factor	C
56547	S-protein homologue	35.2, not assigned	C
33492	Phosphatidylglycerophosphate synthase	11.3.3, lipid metabolism.phospholipid synthesis.phosphatidate cytidyltransferase	C
75387	Ubiquitin protein ligase	29.5.11.4.1, protein.degradation. ubiquitin.E3.HECT	C
95340	Unknown function	35.2, not assigned	C
49446	DELLA protein GAI	35.2, not assigned	C
19729	Dihydroneopterin aldolase	25.9, C1 metabolism.dihdroneopterin aldolase	C
67863	Unknown function	35.2, not assigned	C
11139	Dienelactone hydrolase family	26.1, misc.misc	C
29363	Tryptophan synthase β chain	13.1.6.5.5, aa metabolism.synthesis.aromatic aa.tryptophan.tryptophan synthase	C
51049	Cinnamoyl co-A reductase	16.8.3, secondary metabolism.flavonoids.dihydroflavonols	C
81484	Unknown function	35.2, not assigned	C
58336	Flavonoid-3-O-glycosyltransferase	26.2, misc.UDP glycosyltransferase	D
51992	LRR-receptor kinase	30.2.11, signalling.receptor kinases.leucine rich repeat XI	D
03892	Pollen coat	35.2, not assigned	D
20062	Sn-1 protein	35.2, not assigned	D
27856	Methylesterase	26.8, misc.nitrilases	D
11067	Unknown function	35.2, not assigned	D
66405	Methylesterase	26.8, misc.nitrilases	D
87465	Unknown function	35.2, not assigned	D
88493	β-Glucosidase	26.3, misc.gluco- galacto- and mannosidases	D
92306	Non-specific lipid transfer protein	35.2, not assigned	D
46193	Pectinesterase	10.8.1, cell wall.pectin esterases.PME	D
59754	Sn-2 protein	35.2, not assigned	D
41989	2-Oxoglutarate dependent dioxygenase	17.5.1, hormone.ethylene.synthesis-degradation	D
19638	Unknown function	35.2, not assigned	D

Transcripts are listed in the order indicated in **Figure 7** and are identified by their transcript accession (transcript ID) which for clarity omits the common PMT4000 prior to the specific accession ID. A brief description is provided along with the MapMan bin number and descriptive bin identifier. Group refers to the group identified in **Figure 7**.

The final group of transcripts exhibited greater abundance in abiotic stress resistant genotypes than stress sensitive genotypes both under control and stress conditions (**Figure 7D**). This group included transcripts (DMT400058336, DMT400051992) homologous to an *Arabidopsis* plastid localized flavonoid-3-O-glycosyltransferase (At5g65550) and a mitochondrially localized leucine rich repeat receptor kinase (At1g03440, **Table 1**). Several transcripts were associated with hormone metabolism and signal transduction with two methylesterases (DMT400027856, DMT400066405) exhibiting homology to *Arabidopsis* transcripts (At3g50440, At2g23610) shown to demethylate jasmonate and auxin *in vitro* and a transcript encoding a 2-oxoglutarate dependent dioxygenase (DMT400041989) homologous to an *Arabidopsis* transcript (AtAt1g06620) involved in drought and ethylene responses (Manavella et al., 2006).

### Metabolic Profiles of Potato Leaves Subjected to Single and Combined Abiotic Stress

GC/MS was used to determine primary metabolite profiles in leaves of the four potato genotypes under control and stress treatments. Samples were extracted with phase separation allowing analysis of both polar and non-polar metabolites. A total of 104 chromatogram peaks were quantified against appropriate internal standards of which 66 were present in the polar extract and 38 in the non-polar extract.

**Figure 8:**
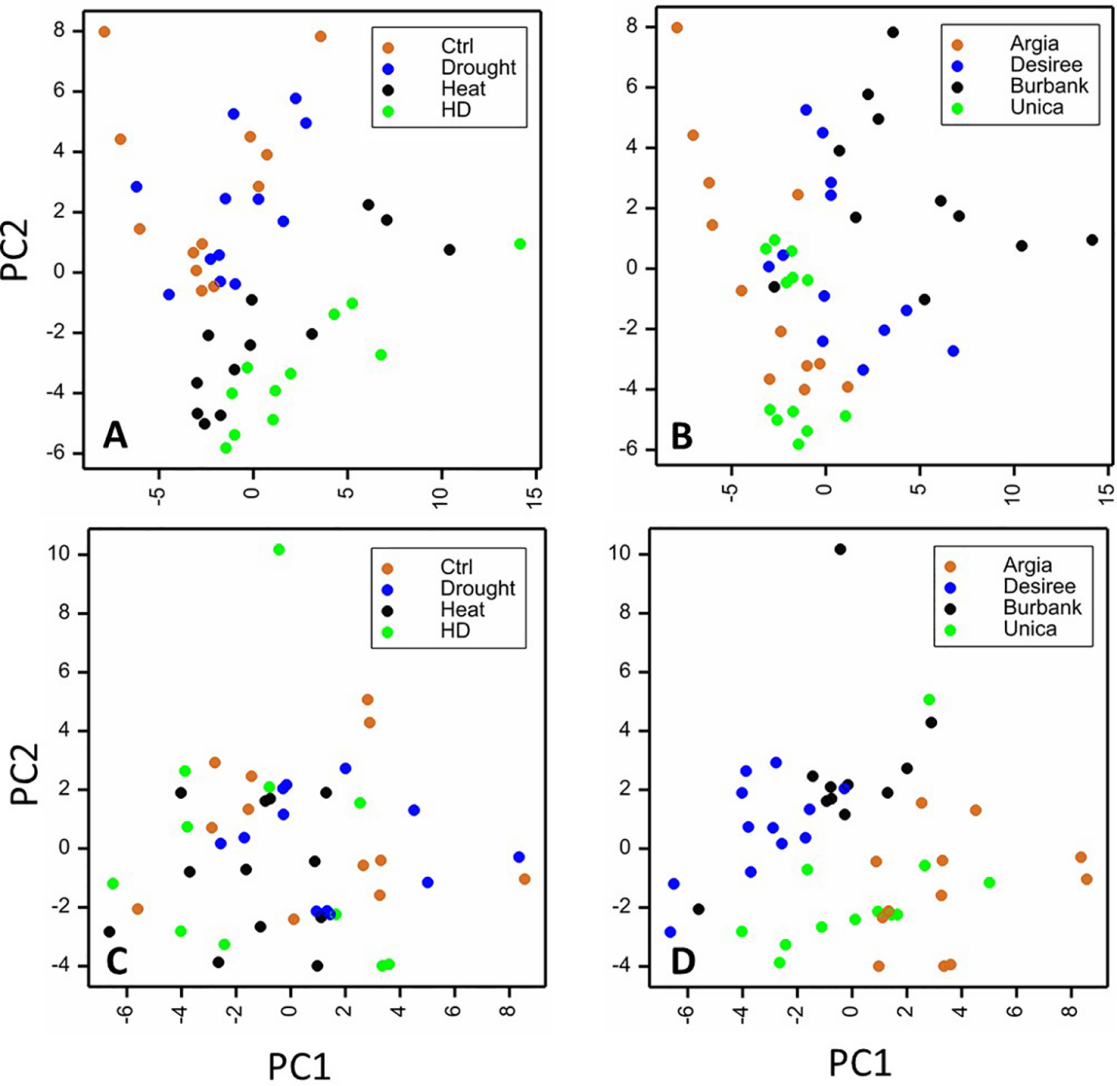
Principal components analysis (PCA) plots of leaf samples based on metabolite profiles. Sample replicates are plotted against principal components (PC) 1 and 2 based on their content of polar **(A**, **B)** or non-polar **(C**, **D)** metabolites. Samples are labeled according to treatment **(A**, **C)** or genotype **(B**, **D)**. For polar compounds PCs 1 and 2 represented 27 and 18% of the variance, respectively and for non-polar compounds PC1 represented 30% of the variability while PC2 represented 20%.

To obtain an overview of the influence of cultivar and treatment on the leaf metabolome, PCA analyses were undertaken on the polar and non-polar fractions independently. When considering the polar metabolites, samples clearly clustered dependent on heat treatment (heat or drought and heat) (**Figure 8A**). Principal component 2 (representing 19% of the variation) was the primary driver for differences between treatments. There was also clustering dependent on genotype, with the primary differences being driven by principal component 1 representing 27% of the variation (**Figure 8B**). When samples were plotted dependent on their non-polar metabolite profiles, clustering of samples was less clear, particularly when treatment was considered (**Figure 8C**) although genotypes did exhibit a degree of clustering that was primarily dependent on principal component 1 representing 30% of the variation in the dataset (**Figure 8D**).

**Figure 9:**
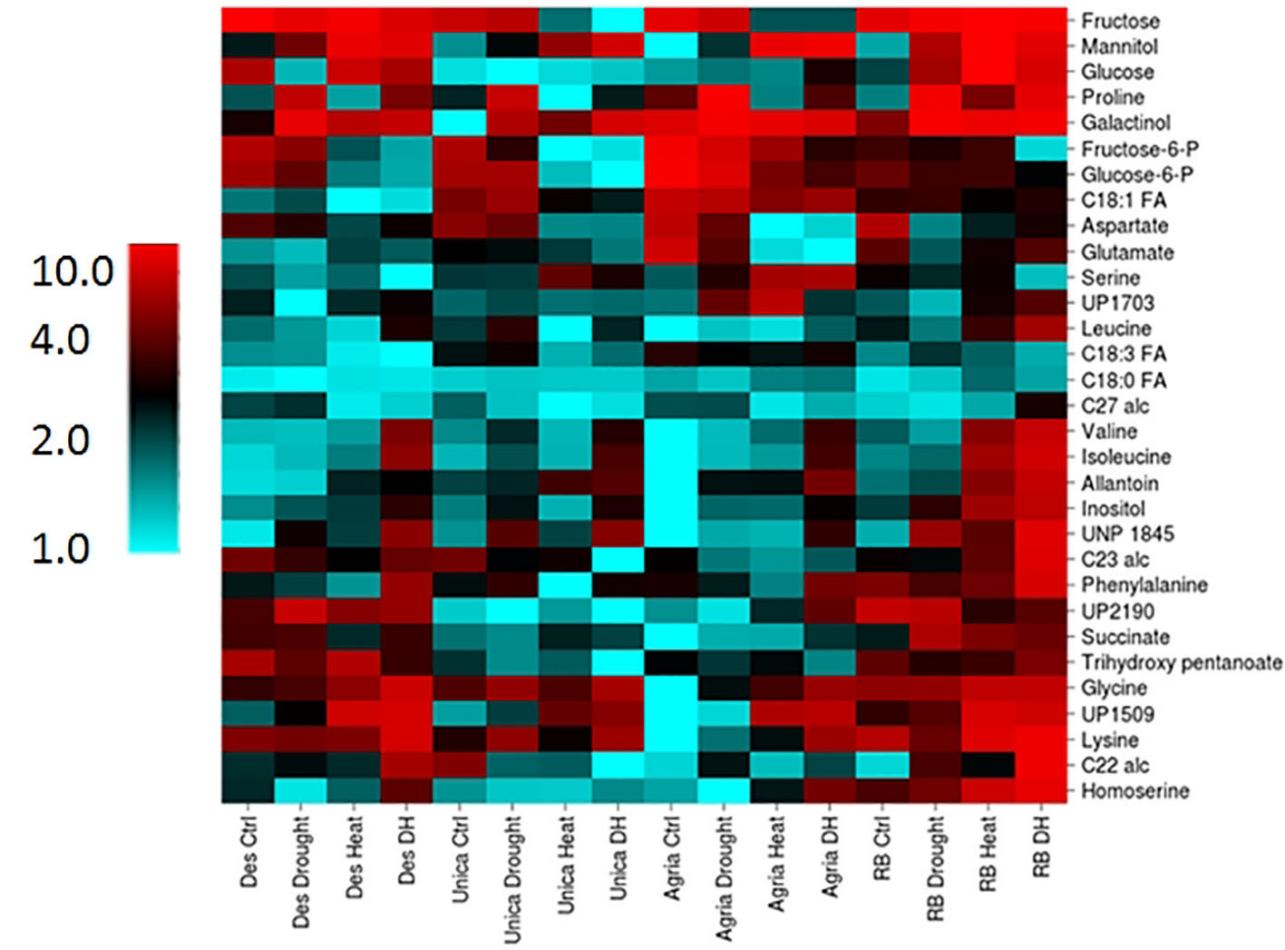
Heatmap of leaf metabolites exhibiting a genotype x treatment interactive effect on concentration. Columns labels indicate genotype and treatment. Metabolites were clustered using the CIMminer tool ((https://discover.nci.nih.gov/cimminer/home.do, Weinstein et al., 1994) following normalization by calculating the mean abundance of each metabolite from three independent biological replicates relative to the sample in which each metabolite had the lowest mean concentration.

Two-way analysis of variance revealed a total of 82 components that were significantly different between genotypes and 65 that were significantly altered by stress treatment (**Supplementary Table S3**). Thirty-one components comprising 24 polar and 7 non-polar compounds exhibited a genotype by treatment interaction and these were selected for further analysis to identify metabolic changes commonly associated with stress tolerance or sensitivity. Data were clustered and represented using the CIMminer tool (https://discover.nci.nih.gov/cimminer/home.do, Weinstein et al., 1994). This analysis did not reveal any clusters of metabolites exhibiting similar behavior in either the stress resistant or the stress susceptible cultivars (**Figure 9**). However, a more detailed analysis of the data did reveal some interesting genotype specific changes in metabolite profiles in response to stress.

Among the sugar alcohols, galactinol concentration increased in all genotypes in response to drought while inositol and mannitol were more responsive to heat and drought and heat treatments (**Supplementary Figure S4**). Proline was strongly accumulated following drought in the stress susceptible cultivars Agria and Russett Burbank however combined heat and drought suppressed this accumulation relative to drought alone (**Supplementary Figure S4**). On the contrary, the combined heat and drought treatment resulted in the highest accumulation of Valine, isoleucine, and lysine in all genotypes (**Supplementary Figure S4**). Allantoin is a purine ring catabolite involved in nitrogen remobilization and protection from abiotic stress (Werner and Witte, 2011). Allantoin concentration increased in leaves of all genotypes following heat or combined heat and drought stress (**Supplementary Figure S4**).

## Discussion

Drought and heat are the major abiotic stresses affecting marketable potato tuber yield (Monneveux et al., 2013; Aksoy et al., 2015) and often occur simultaneously in prone regions leading to further crop losses (Mittler, 2006; Zandalinas et al., 2018). In recent years many studies have focused on the impact of heat or drought stress in isolation in potato (Hancock et al., 2014; Monneveux et al., 2013; Sprenger et al., 2016; Sprenger et al., 2018) however, reports concerning combined heat and drought stress are limited (Rensink et al., 2005). Studies in other species have shown that data from single stress experiments cannot be used to predict the tolerance to multiple stress situations (Prasch and Sonnewald, 2015). Rizhsky et al. (2002, 2004) investigated combined heat and drought stress in tobacco and *Arabidopsis* and observed that under these stress conditions plants were unable to open their stomata, a process that would normally occur under heat stress to aid cooling *via* transpiration. Combined heat and drought stress resulted in accumulation of specific metabolites and transcripts that were unique and not present in the single stress treated plants. Drought and heat tolerance, and combination thereof, are complex traits that make resistance breeding a challenge. Understanding the unique molecular and metabolic responses to combined abiotic stresses will help to facilitate breeding for tolerant cultivars. In this study we used a combined physiological, transcriptomic, and metabolomic approach to characterize the response of potato cultivars with contrasting abiotic stress tolerance to identify transcript and metabolite markers for potential use in future breeding programs.

The initial aim of the work was to determine stress conditions that differentiate between the stress tolerant cultivars Desiree and Unica and the stress sensitive cultivars Agria and Russet Burbank. As photosynthetic assimilation and transport to support developing tubers are key factors influencing yield under stress (Reynolds et al., 1990; Wolf et al., 1991; Hancock et al., 2014), we chose to monitor stress induction by quantification of photosynthetic assimilation. These experiments highlighted the much greater inhibition of photoassimilation in the sensitive cultivars than in the tolerant cultivars following 12 days of stress (**Figure 2**). Clear and differential impacts on plant morphology were also observed at this time point (**Figure 1**) and hence it was chosen for our subsequent analysis.

In addition to the reported detrimental effects of stress interaction on plant growth and crop yield some studies have reported a positive impact (Zandalinas et al., 2018). However, the results from this study show a clear negative effect of heat and drought interaction on potato foliage development and photosynthetic performance under two levels of stress severity. Sensitive cultivars exhibited a greater reduction in net carbon assimilation compared to tolerant cultivars suggesting that tolerance may be due in part to enhanced photosynthetic capacity. A similar study in lentil (Sehgal et al., 2017) observed a significantly greater reduction in net photosynthesis under drought stress than heat stress alone. However, under our experimental conditions we did not observe any significant difference between individual heat and drought stress on Pn although drought, heat, and combined stress severely restricted leaf transpiration in all cultivars.

Photosynthetic limitation is often accompanied by the accumulation of reactive oxygen species in the thylakoid stroma which can be detoxified by the action of plastid localized SOD and APX (Foyer et al., 2020). We failed to observe any changes in total SOD activity in leaves following the imposition of abiotic stress in any of the cultivars, however as SOD is present in multiple cellular compartments (Hancock, 2017) an increase in plastid localized activity may have been masked by changes in activity in other cellular compartments. APX activity was enhanced by stress treatment for most of the cultivars although no significant changes were observed in the case of Agria. On the contrary, CAT activity was decreased by stress treatment in most cultivars with the exception of Agria where it was increased (**Figure 5**). Given that catalase has a much lower affinity for H_2_O_2_ than APX (Km approximately 1,000 times higher) and that catalase is primarily localized to the peroxisomes while APX is found in most cellular compartments (Hancock, 2017) these data suggest that Agria may experience a greater oxidative burden than the other cultivars under conditions of abiotic stress, particularly as unlike the other cultivars POD activity was not enhanced by stress treatment in Agria. However, analysis of malondialdehyde, a marker for ROS accumulation, revealed no significant differences between control and stressed plants in Agria or any other cultivars with the exception of Russet Burbank that exhibited an increase only under drought stress. It therefore appears that despite the differences in severity of photosynthetic inhibition and antioxidant enzyme activity, severe oxidative damage was avoided in all cultivars. However, it is also possible that plants experienced severe oxidative stress at an earlier time point in treatment that resulted in the induction of antioxidant systems and repair mechanisms that were not observed in our experiments due to our necessary focus on the 12 day time point. These data raise the possibility that at the 12 day time point, plants may no longer be in an acute stress phase and may have entered an acclimatory phase. Future experiments will need to examine the dynamics of biochemical changes more closely to fully resolve stress and acclimatory phases.

Analysis of the leaf metabolome by GC/MS indicated potential metabolites that may have contributed to protection against oxidative damage. The osmoprotectant proline (considered to be important for plant protection during drought stress; Ashraf and Foolad, 2007) significantly increased under drought stress as estimated by both spectrophotometric determination using a ninhydrin reagent and untargeted GC/MS. Spectrophotometric determination indicated that proline accumulation was impaired in combined drought and heat stress in Agria and Unica but was maintained in Russet Burbank and Desiree. However, the spectrophotometric method is prone to interference by other amino acids (Bates et al., 1973) and sugars (Magné and Larher, 1992) and in our experiments GC/MS suggested that proline accumulation was strongly suppressed in all genotypes following heat and drought stress when compared with drought alone (**Supplementary Figure S4**). These data confirm previous results in *Arabidopsis* where a strong reduction in proline accumulation was observed under combined heat and drought stress (Rizhsky et al., 2004). Comparisons of tolerant and sensitive cultivars revealed accumulation of specific metabolites in sensitive cultivars under combined stress including certain amino acids reducing sugars and polyols. Although accumulation of polyols in plants subjected to abiotic stress has previously been reported for apple, barley and potato (Chen et al., 2007; Sircelj et al 2005; Sprenger et al., 2016; Drapal et al., 2017), further work is required to understand the functional significance of the accumulation of these compounds only in the stress sensitive cultivars.

Transcriptomic analysis demonstrated considerable overlap between our data set and those from similar experiments published previously. We compared expression profiles of transcripts in our datasets with previous studies that were focused on transcript profiles in drought (Pieczynski et al., 2018) or heat (Gangadhar et al., 2014) stressed leaves. Our datasets exhibited extensive overlap with previous work where 18 of 23 drought-associated transcripts and 45 of 65 heat-associated transcripts were differentially expressed in our dataset (**Supplementary Figures S2** and **S3**). Many of these transcripts were even more highly expressed in the combined drought and heat stress than in the respective drought or heat treatments alone. A key question that remains is whether these transcript profiles are associated with acute stress responses or with acclimatory responses that allow plants to survive until more favorable environmental conditions return. Although our physiological data demonstrated severe photosynthetic restriction, biochemical markers of stress were less pronounced with almost no elevation in leaf MDA content. These data suggest that leaves may have entered an acclimatory phase that limited oxidative damage by day 12 following stress. Our enzyme activity measurements suggest that this may have partially been achieved by elevation of antioxidant capacity although the observation that combined heat and drought tended to suppress some antioxidant enzymes without causing an increase in oxidative stress markers suggests that mechanisms other than those quantified may be partly responsible for limiting oxidative damage or upregulating repair.

In order to differentiate specific transcriptome signatures that may impart stress tolerance or sensitivity, we subsequently focused our analysis on a set of 461 transcripts that showed genotype-dependent expression changes in response to stress. Our MapMan and PageMan analyses are suggestive of a dynamic, genotype dependent signaling program in response to abiotic stress.

Desiree and Unica (heat tolerant) both exhibited a significant reduction in the abundance of transcripts associated with PSII, particularly transcripts encoding light harvesting chlorophyll-protein complex components, when exposed to drought and heat and no such reduction was observed in Agria or Russett Burbank. Similarly, a strong reduction in these transcripts was observed under conditions of high temperature alone in Desiree (**Figure 6**). These data suggest that tolerant cultivars acclimate to stress by reducing their light harvesting capacity thereby minimizing the potential for cellular damage resulting from the production of excessive ROS in the photosynthetic electron transport chain. Similarly, only the stress tolerant genotypes exhibited an elevated leaf temperature under heat and drought stress in comparison with heat alone. This would be consistent with the induction of non-photochemical quenching of chlorophyll fluorescence and the increased radiation of absorbed light energy as heat (Ruban, 2016).

A sub-set of 54 transcripts showed opposite patterns of expression in both sensitive varieties compared with both tolerant varieties. We reason that these genes are candidates for having important roles in stress response or acclimation that could underpin tolerance or sensitivity. While the detailed function of many transcripts in this set remains to be resolved, several already offer insights into stress response mechanisms. One interesting example is a transcript (DMT400036544) with homology to an *Arabidopsis* transcript encoding ABI5 binding protein 2 (AFP2, At1g13740). The transcription factor ABI5 is a key regulator of ABA signaling and stress responses and its function is modulated by AFP2 (Garcia et al., 2008). This provides a mechanism for fine-tuning stress responses and so its differential expression between heat tolerant and heat sensitive potato varieties is highly significant. Of further potential importance is that this sub-set also contains a DELLA transcript. required for gibberellin signaling in response to environmental signals (Alvey and Harberd, 2005). GA-mediated signaling exhibits crosstalk with other phytohormones including abscisic acid and auxin and hence integrates multiple hormone signaling cascades in response to abiotic stress (Banerjee and Roychoudhury, 2019). Also in this group of transcripts is one encoding a NF-Y transcription factor. Transgenic over-expression of a NF-Y transcription factor in maize was clearly associated with tolerance to drought (Nelson et al., 2007) and so this is a further example of the utility of our dataset.

Interestingly, a series of transcripts associated with metabolic processes were more highly abundant in stress tolerant compared with stress sensitive genotypes (**Figures 7C, D**; **Table 1**). Although the precise functions of some of the encoded proteins are not fully elucidated, the finding that transcripts associated with primary metabolic processes such as sugar and lipid metabolism are among this set of genes indicates that stress tolerant genotypes may have greater capacity to adjust their metabolism ensuring a consistent supply of reducing equivalents and ATP under conditions of reduced photosynthesis.

Aside from the mechanistic insights gained from our transcriptomic dataset analysis, this information is also of value in genetic studies to identify sources of abiotic stress tolerance in potato germplasm, as it will facilitate the identification of candidate genes within QTL limits or associated with other genetic markers.

Developmental stage and severity of stress are important considerations when deciding on abiotic stress treatments and is therefore a limiting factor of any study. We chose to impart stress treatments at the tuber initiation stage as this has been shown to limit foliage, stolon and tuber development and yield (Obidiegwu et al., 2015). To minimize the effects of genotype × environment interaction and diurnal cycle on transcript/metabolite profiles, plants were grown in controlled environment chambers and all sampled at the same time of day. Future experiments should consider verifying the expression patterns of candidate genes over a larger range of accessions with varying tolerance and would help to confirm the potential of putative candidate genes. Further work is also required to identify gene function during stress induction to elucidate the specific transcripts associated with long term acclimatory responses that allow plants to withstand periods of stress and allow the rapid induction of normal metabolism to favorable abiotic conditions.

In summary, we identified unique changes in transcripts and metabolites that were specific to individual and combined heat and drought stresses. We define conditions that highlight differences in sensitivity between different potato genotypes. These experiments indicate that stress tolerant cultivars respond to stress by i) reducing light harvesting capacity and increasing non-photochemical quenching and ii) maintaining capacity for growth and development in part by iii) rerouting metabolism to compensate for reduced photosynthesis. This is achieved by a fine tuning of hormonal signaling. The responses of cultivars with contrasting abiotic stress tolerance provided information on genes/classes of compounds that may be used as targets for future studies aimed at enhancing multi-stress tolerance in potato.

## Data Availability Statement

The datasets generated for this study can be found in the http://www.ebi.ac.uk/arrayexpress/accession number E-MTAB-8298.

## Author Contributions

UD, WM, MT and RH conceived and designed the study. UD, CY, AA, IT, ZG, SC, EA, MC conducted physiological and biochemical experiments. UD, CY, IT, WM and LD prepared material for transcriptome and metabolome analysis. JM conducted microarray experiments that were analysed by PH. WM and RC conducted and analysed metabolite profiling experiments. SV conducted statistical analysis. UD, WM, MT and RH drafted the manuscript with contributions from all authors.

## Funding

This research was funded by a British Council Newton Fund Institutional Links Grant No. 216394957 and the Scottish Government Rural and Environment Science and Analytical Services Division as part of the Strategic Research Programme 2016-2021.

## Conflict of Interest

The authors declare that the research was conducted in the absence of any commercial or financial relationships that could be construed as a potential conflict of interest.

## References

[B1] Aebi H. (1984). “Catalase in Vitro,” in Methods in Enzymology, Ed. Packer L. (San Diego, CA: Academic Press), vol. 105 121–126. 10.1016/s0076-6879(84)05016-3 6727660

[B2] Ahn Y. J. Claussen K. Zimmerman J. L. (2004). Genotypic differences in the heat-shock response and thermotolerance in four potato cultivars. Plant Sci. 166, 901–911. 10.1016/j.plantsci.2003.11.027

[B3] Aksoy E. Demirel U. Ozturk Z. N. Caliskan S. Caliskan M. E. (2015). Recent advances in potato genomics, transcriptomics, and transgenics under drought and heat stress: a review. Turkish J. Bot. 39, 920–940. 10.3906/bot-1506-25

[B4] Alvey L. Harberd N. P. (2005). DELLA proteins: integrators of multiple plant growth regulatory inputs? Physiol. Plant 123, 153–160. 10.1111/j.1399-3054.2004.00412.x

[B5] Ashraf M. Foolad M. R. (2007). Roles of glycine betaine and proline in improving plant abiotic stress resistance. Env. Exp. Bot. 59, 206–216. 10.1016/j.envexpbot.2005.12.006

[B6] Banerjee A. Roychoudhury A. (2019). “The regulatory signaling of gibberellin metabolism and its crosstalk with phytohormones in response to plant abiotic stresses,” in Plant signaling molecules (Cambridge, UK: Woodhead Publishing), 333–339.

[B7] Basu P. S. Minhas J. S. (1991). Heat tolerance and assimilate transport in different potato genotypes. J. Exp. Bot. 42, 861–866. 10.1093/jxb/42.7.861

[B8] Bates L. S. Waldren R. P. Teare I. D. (1973). Rapid determination of free proline for water-stress studies. Plant Soil 39, 205–207. 10.1007/BF00018060

[B9] Bejarano L. Mignolet E. Devaux A. Espinola N. Carrasco E. Larondelle Y. (2000). Glycoalkaloids in potato tubers: the effect of variety and drought stress on the α-solanine and α-chaconine contents of potatoes. J. Sci. Food Agric. 80, 2096–2100. 10.1002/1097-0010(200011)80:14<2096::AID-JSFA757>3.0.CO;2-6

[B10] Benjamini Y. Yekutieli D. (2001). The control of the false discovery rate in multiple testing under dependency. Annal. Stat. 29, 1165–1188. 10.1214/aos/1013699998

[B11] Birch P. R. J. Bryan G. Fenton B. Gilroy E. M. Hein I. Jones J. T. (2012). Crops that feed the world 8: potato: are the trends of increased global production sustainable? Food Secur. 4, 477–508. 10.1007/s12571-012-0220-1

[B12] Chen Z. Cuin T. A. Zhou M. Twomey A. Naidu B. P. Shabala S. (2007). Compatible solute accumulation and stress-mitigating effects in barley genotypes contrasting in their salt tolerance. J. Exp. Bot. 58, 4245–4255. 10.1093/jxb/erm284 18182428

[B13] Coll N. S. Vercammen D. Smidler A. Clover C. Van Breusegem F. Dangl J. L. (2010). Arabidopsis type I metacaspases control cell death. Science 330, 1393–1397. 10.1126/science.1194980 21097903

[B14] Deblonde P. M. K. Ledent J. F. (2001). Effects of moderate drought conditions on green leaf number, stem height, leaf length and tuber yield of potato cultivars. Eur. J. Agron. 14, 31–41. 10.1016/S1161-0301(00)00081-2

[B15] Demirel U. Çaliskan S. Yavus C. Tindas I. Polgar Z. Vaszily Z. (2017). Assessment of morphophysiological traits for selection of heat-tolerant potato genotypes. Turkish J. Agric. For. 41, 218–232. 10.3906/tar-1701-95

[B16] Dobson G. Shepherd T. Verrall S. R. Conner S. McNicol J. W. Ramsay G. (2008). Phytochemical diversity in tubers of potato cultivars and landraces using a GC-MS metabolomics approach. J. Agric. Food Chem. 56, 10280–10291. 10.1021/jf801370b 18937493

[B17] Drapal M. Farfan-Vignolo E. R. Gutierrez O. R. Bonierbale M. Mihovilovich E. Fraser P. D. (2017). Identification of metabolites associated with water stress responses in *Solanum tuberosum* L. clones. Phytochemistry 135, 24–33. 10.1016/j.phytochem.2016.12.003 27964835

[B18] Ducreux L. J. M. Morris W. L. Prosser I. M. Morris J. A. Beale M. H. Wright F. (2008). Expression profiling of potato germplasm differentiated in quality traits leads to the identification of candidate flavour and texture genes. J. Exp. Bot. 59, 4219–4231. 10.1093/jxb/ern264 18987392 PMC2639024

[B19] Echevarría-Zomeño S. Fernández-Calvino L. Castro-Sanz A. B. López J. A. Vázquez J. Castellano M. M. (2016). Dissecting the proteome dynamics of the early heat stress response leading to plant survival or death in Arabidopsis. Plant Cell Environ. 39, 1264–1278. 10.1111/pce.12664 26580143

[B20] Eiasum B. K. Soundy P. Hammes P. S. (2007). Response of potato (*Solanum tuberosum*) tuber yield components to gel polymer soil amendments and irrigation regimes. N. Z. J. Crop Hortic. 35, 25–31. 10.1080/01140670709510164

[B21] Evers D. Lefèvre I. Legay S. Lamoureux D. Hausman J. F. Rosales R. O. (2010). Identification of drought-responsive compounds in potato through a combined transcriptomic and targeted metabolite approach. J. Exp. Bot. 61, 2327–2343. 10.1093/jxb/erq060 20406784

[B22] Foito A. Byrne S. L. Hackett C. A. Hancock R. D. Stewart D. Barth S. (2013). Short-term response in leaf metabolism of perennial ryegrass (*Lolium perenne*) to alterations in nitrogen supply. Metabolomics 9, 145–156. 10.1007/s11306-012-0435-3

[B23] Foyer C. H. Kyndt T. Hancock R. D. (2020). Vitamin C in plants: novel concepts, new perspectives and outstanding issues. Antioxid. Redox. Signal. 32, 463–485. 10.1089/ars.2019.7819 31701753

[B24] Gangadhar B. H. Yu J. W. Sajeesh K. Park S. W. (2014). A systematic exploration of high-temperature stress-responsive genes in potato using large-scale yeast functional screening. Mol. Genet. Genomics 289, 185–201. 10.1007/s00438-013-0795-z 24357347

[B25] Garcia M. E. Lynch T. Peeters J. Snowden C. Finkelstein R. (2008). A small plant-specific protein family of ABI five binding proteins (AFPs) regulates stress response in germinating Arabidopsis seeds and seedlings. Plant Mol. Biol. 67, 643–658. 10.1007/s11103-008-9344-2 18484180

[B26] Giannopolitis C. N. Ries S. K. (1977). Superoxide dismutases: I. Occurrence in higher plants. Plant Physiol. 59, 309–314. 10.1104/pp.59.2.309 16659839 PMC542387

[B27] Gregory L. E. (1965). “Physiology of tuberization in plants. (Tubers and tuberous roots.),” in Differentiation and development. Encyclopedia of plant physiology, Ed. Lang A. (Berlin, Heidelberg: Springer), vol. 15.

[B28] Gutiérrez-Rosales R. O. Espinoza-Trelles J. A. Bonierbale M. (2007). UNICA: variedad Peruana para mercado fresco y papa frita con tolerancia y resistencia para condiciones climáticas adversas. Rev. Latinoamericana la Papa. 14, 41–50.

[B29] Hancock R. D. Morris W. L. Ducreux L. J. M. Morris J. A. Usman M. Verrall S. R. (2014). Physiological, biochemical and molecular responses of the potato (*Solanum tuberosum* L.) plant to moderately elevated temperature. Plant Cell Environ. 37, 439–450. 10.1111/pce.12168 23889235

[B30] Hancock R. D. (2017). “Oxidative Stress,” in Encyclopedia of Applied Plant Sciences, Eds. Thomas B. Murray B. G. Murphy D. J. (Waltham, MA: Academic Press), vol. 1, 27–35. 10.1016/B978-0-12-394807-6.00139-8

[B31] Harris P. M. (1978). The potato crop — The scientific basis for improvement (London: Chapman and Hall).

[B32] Heath R. L. Packer L. (1968). Photoperoxidation in isolated chloroplast. I. Kinetics and stoichiometry of fatty acid peroxidation. Arch. Biochem. Biophys. 125, 189–198. 10.1016/0003-9861(68)90654-1 5655425

[B33] Hijmans R. J. (2003). The effect of climate change on global potato production. Am. J. Pot. Res. 80, 271–280. 10.1007/BF02855363

[B34] Hsieh E. J. Cheng M. C. Lin T. P. (2013). Functional characterization of an abiotic stress-inducible transcription factor AtERF53 in Arabidopsis thaliana. Plant Mol. Biol. 82, 223–237. 10.1007/s11103-013-0054-z 23625358

[B35] Iwama K. Yamaguchi J. (2006). “Abiotic stresses,” in Handbook of potato production, improvement and postharvest management. Eds. Gopal J. Paul K. S. M. (New York: Food Product Press), 231–278.

[B36] Jeffery R. A. (1995). “Physiology of crop response to drought,” in Potato ecology and modelling of crops under conditions limiting growth. Eds. Haverkort A. J. MacKerron D. K. L. (Netherlands: Wageningen Academic Publishers).

[B37] Kabbage M. Kessens R. Bartholomay L. C. Williams B. (2017). The life and death of a plant cell. Ann. Rev. Plant Biol. 68, 375–404. 10.1146/annurev-arplant-043015-111655 28125285

[B38] Kaur G. Asthir B. (2015). Proline: a key player in plant abiotic stress tolerance. Biol. Plant 59, 609–619. 10.1007/s10535-015-0549-3

[B39] Kilian J. Peschke F. Berendzen K. W. Harter K. Wanke D. (2012). Prerequisites, performance and profits of transcriptional profiling the abiotic stress response. Biochim. Biophys. Acta 1819, 166–175. 10.1016/j.bbagrm.2011.09.005 22001611

[B40] Lehretz G. G. Sonnewald S. Hornyik C. Corral J. M. Sonnewald U. (2019). Post-transcriptional regulation of FLOWERING LOCUS T modulates heat-dependent source-sink development in potato. Curr. Biol. 29, 1614–1624. 10.1016/j.cub.2019.04.027 31056391

[B41] Levy D. Veilleux R. E. (2007). Adaptation of potato to high temperatures and salinity - a review. Am. J. Pot. Res. 84, 487–506. 10.1007/BF02987885

[B42] Maehly A. C. Chance B. (1954). “The assay of catalases and peroxidases,” in Methods of biochemical analysis, Ed. Glick D., (New York, USA: Interscience Publishers), vol. 1 357–424. 10.1002/9780470110171.ch14 13193536

[B43] Magné C. Larher F. (1992). High sugar content of extracts interferes with colorimetric determination of amino acids and free proline. Anal. Biochem. 200, 115–118. 10.1016/0003-2697(92)90285-F 1595885

[B44] Manavella P. A. Arce A. L. Dezar C. A. Bitton F. Renou J.-P. Crespi M. (2006). Cross-talk between ethylene and drought signalling pathways is mediated by the sunflower Hahb-4 transcription factor. Plant J. 48, 125–137. 10.1111/j.1365-313X.2006.02865.x 16972869

[B45] Martínez C. A. Moreno U. (1992). Expresiones fisiológicas de resistencia a sequía en dos variedades de papa sometidas a estrés hídrico. Rev. Bras. Fisiol. Veget. 4, 33–38.

[B46] Miao Y. Zentgraf U. (2010). A HECT E3 ubiquitin ligase negatively regulates Arabidopsis leaf senescence through degradation of the transcription factor WRKY53. Plant J. 63, 179–188. 10.1111/j.1365-313X.2010.04233.x 20409006

[B47] Mittler R. (2006). Abiotic stress, the field environment and stress combination. Trends Plant Sci. 11, 15–19. 10.1016/j.tplants.2005.11.002 16359910

[B48] Monneveux P. Ramírez D. A. Pino M.-T. (2013). Drought tolerance in potato (*S. tuberosum* L.): can we learn from drought tolerance research in cereals? Plant Sci. 205–206, 76–86. 10.1016/j.plantsci.2013.01.011 23498865

[B49] Morris W. L. Hancock R. D. Ducreux L. J. M. Morris J. A. Usman M. Verrall S. R. (2014). Day length dependent restructuring of the leaf transcriptome and metabolome in potato genotypes with contrasting tuberization phenotypes. Plant Cell Environ. 37, 1351–1363. 10.1111/pce.12238 24236539

[B50] Morris W. L. Ducreux L. J. Morris J. Campbell R. Usman M. Hedley P. E. (2019). Identification of TIMING OF CAB EXPRESSION 1 as a temperature-sensitive negative regulator of tuberisation in potato. J. Exp. Bot. 70, 5703–5714. 10.1093/jxb/erz336 31328229 PMC6812706

[B51] Nakano Y. Asada K. (1981). Hydrogen peroxide is scavenged by ascorbate-specific peroxidase in spinach chloroplasts. Plant Cell Physiol. 22, 867–880. 10.1093/oxfordjournals.pcp.a076232

[B52] Nelson D. E. Repetti P. P. Adams T. R. Creelman R. A. Wu J. Warner D. C. (2007). Plant nuclear factor Y (NF-Y) B subunits confer drought tolerance and lead to improved corn yields on water-limited acres. Proc. Natl. Acad. Sci. U. S. A. 104, 16450–16455. 10.1073/pnas.0707193104 17923671 PMC2034233

[B53] Nie L. Z. Yu X. X. Ma Y. H. Fang Y. Y. Li L. M. Yu Z. (2018). Enhanced drought and osmotic stress tolerance in transgenic potato plants expressing AtCDPK1, a calcium-dependent protein kinase. Russian J. Plant Physiol. 65, 865–873. 10.1134/S1021443718060110

[B54] Noctor G. Foyer C. H. (1998). Ascorbate and glutathione: keeping active oxygen under control. Ann. Rev. Plant Physiol. Plant Mol. Biol. 49, 249–279. 10.1146/annurev.arplant.49.1.249 15012235

[B55] Obidiegwu J. E. Bryan G. J. Jones H. G. Prashar A. (2015). Coping with drought: stress and adaptive responses in potato and perspectives for improvement. Front. Plant Sci. 6, 542. 10.3389/fpls.2015.00542 26257752 PMC4510777

[B56] Pan Y. J. Liu L. Lin Y. C. Zu Y. G. Li L. P. Tang Z. H. (2016). Ethylene antagonizes salt-induced growth retardation and cell death process *via* transcriptional controlling of ethylene-, BAG- and senescence-associated genes in Arabidopsis. Front. Plant Sci. 7, 696. 10.3389/fpls.2016.00696 27242886 PMC4872043

[B57] Per T. S. Khan N. A. Reddy P. S. Masood A. Hasanuzzaman M. Khan I. R. (2017). Approaches in modulating proline metabolism in plants for salt and drought stress tolerance: phytohormones, mineral nutrients and transgenics. Plant Physiol. Biochem. 115, 126–140. 10.1016/j.plaphy.2017.03.018 28364709

[B58] Pieczynski M. Wyrzkowska A. Milanowska K. Boguszewska-Mankowska D. Zagdanska B. Karlowski W. (2018). Genomewide identification of genes involved in the potato response to drought indicates functional and evolutionary conservation with *Arabidopsis* plants. Plant Biotech. J. 16, 603–614. 10.1111/pbi.12800 PMC5787840 28718511

[B59] Prasch C. M. Sonnewald U. (2015). Signaling events in plants: stress factors in combination change the picture. Env. Exp. Bot. 114, 4–14. 10.1016/j.envexpbot.2014.06.020

[B60] Rensink W. Hart A. Liu J. Ouyang S. Zismann V. Buell C. R. (2005). Analyzing the potato abiotic stress transcriptome using expressed sequence tags. Genome 48, 598–605. 10.1139/g05-034 16094426

[B61] Reynolds M. P. Ewing E. E. Owens T. G. (1990). Photosynthesis at high temperature in tuber-bearing Solanum species. Plant Physiol. 93, 791–797. 10.1104/pp.93.2.791 16667538 PMC1062585

[B62] Rizhsky L. Liang H. Mittler R. (2002). The combined effect of drought stress and heat shock on gene expression in tobacco. Plant Physiol. 130, 1143–1152. 10.1104/pp.006858 12427981 PMC166635

[B63] Rizhsky L. Liang H. Shuman J. Shulaev V. Davletova S. Mittler R. (2004). When defense pathways collide: the response of Arabidopsis to a combination of drought and heat stress. Plant Physiol. 134, 1683–1696. 10.1104/pp.103.033431 15047901 PMC419842

[B64] Rolando J. L. Ramírez D. A. Yactayo W. Monneveux P. Quiroz R. (2015). Leaf greenness as a drought tolerance related trait in potato (*Solanum tuberosum* L.). Env. Exp. Bot. 110, 27–35. 10.1016/j.envexpbot.2014.09.006

[B65] Ruban A. V. (2016). Nonphotochemical chlorophyll fluorescence quenching: mechanism and effectiveness in protecting plants from photodamage. Plant Physiol. 170, 1903–1916. 10.1104/pp.15.01935 26864015 PMC4825125

[B66] Schafleitner R. Gutierrez R. Espino R. Gaudin A. Pérez J. Martínez M. (2007). Field screening for variation of drought tolerance in *Solanum tuberosum* L. by agronomical, physiological and genetic analysis. Potato Res. 50, 71–85. 10.1007/s11540-007-9030-9

[B67] Sehgal A. Sita K. Kumar J. Kumar S. Singh S. Siddique K. H. M. (2017). Effects of drought, heat and their interaction on the growth, yield and photosynthetic function of lentil (*Lens culinaris* Medikus) genotypes varying in heat and drought sensitivity. Front. Plant Sci. 8, 1776. 10.3389/fpls.2017.01776 29089954 PMC5651046

[B68] Sircelj H. Tausz M. Grill D. Batic F. (2005). Biochemical responses in leaves of two apple tree cultivars subjected to progressing drought. J. Plant Physiol. 162, 1308–1318. 10.1016/j.jplph.2005.01.018 16425449

[B69] Slater J. W. (1968). The effect of night temperature on tuber initiation of the potato. Eur. Potato J. 11, 14–22. 10.1007/BF02365158

[B70] Sprenger H. Kurowsky C. Horn R. Erban A. Seddig S. Rudack K. (2016). The drought response of potato reference cultivars with contrasting tolerance. Plant Cell Environ. 39, 2370–2389. 10.1111/pce.12780 27341794

[B71] Sprenger H. Erban A. Seddig S. Rudack K. Thalhammer A. Le M. Q. (2018). Metabolite and transcript markers for the prediction of potato drought tolerance. Plant Biotechnol. J. 16, 939–950. 10.1111/pbi.12840 28929574 PMC5866952

[B72] Stark J. C. Love S. L. King B. A. Marshall J. M. Bohl W. H. Salaiz T. (2013). Potato cultivar response to seasonal drought patterns. Am. J. Potato Res. 90, 207–216. 10.1007/s12230-012-9285-9

[B73] Suzek B. E. Wang Y. Huang H. McGarvey P. B. Wu C. H. (2015). UniProt Consortium. UniRef clusters: a comprehensive and scalable alternative for improving sequence similarity searches. Bioinformatics 31, 926–932. 10.1093/bioinformatics/btu739 25398609 PMC4375400

[B74] The Potato Genome Consortium (2011). Genome sequence and analysis of the tuber crop potato. Nature 475, 189–195. 10.1038/nature10158 21743474

[B75] Thiele G. Theisen K. Bonierbale M. Walker T. (2010). Targeting the poor and hungry with potato science. Potato J. 37, 75–86.

[B76] Thimm O. Bläsing O. Gibon Y. Nagel A. Meyer S. Krüger P. (2004). Mapman: a user-driven tool to display genomics data sets onto diagrams of metabolic pathways and other biological processes. Plant J. 37, 914–939. 10.1111/j.1365-313X.2004.02016.x 14996223

[B77] Trapero-Mozos A. Ducreux L. J. M. Bita C. E. Morris W. L. Weise C. Morris J. A. (2018a). A reversible light− and genotype−dependent acquired thermotolerance response protects the potato plant from damage due to excessive temperature. Planta 247, 1377–1392. 10.1007/s00425-018-2874-1 29520461 PMC5945765

[B78] Trapero-Mozos A. Morris W. L. Ducreux L. J. M. McLean K. Stephens J. Torrance L. (2018b). Engineering heat tolerance in potato by temperature-dependent expression of a specific allele of HEAT-SHOCK COGNATE 70. Plant Biotechnol. J. 16, 197–207. 10.1111/pbi.12760 28509353 PMC5785350

[B79] Ullah A. Manghwar H. Shaban M. Khan A. H. Akbar A. Ali U. (2018). Phytohormones enhanced drought tolerance in plants: a coping strategy. Environ. Sci. Pollut. Res. Int. 25, 33103–33118. 10.1007/s11356-018-3364-5 30284160

[B80] Usadel B. Nagel A. Steinhauser D. Gibon Y. Blasing O. E. Redestig H. (2006). PageMan: an interactive ontology tool to generate, display, and annotate overview graphs for profiling experiments. BMC Bioinf. 7, 535. 10.1186/1471-2105-7-535 PMC1766370 17176458

[B81] van Loon C. D. (1981). The effect of water stress on potato growth, development, and yield. Am. Pot. J. 58, 51–69. 10.1007/BF02855380

[B82] Weinstein J. N. Myers T. Buolamwini J. Raghavan K. Van Osdol W. Licht J. (1994). Predictive statistics and artificial intelligence in the U.S. National Cancer Institute's drug discovery program for cancer and AIDS. Stem Cells 12, 13–22. 10.1002/stem.5530120106 8142917

[B83] Werner A. K. Witte C.-P. (2011). The biochemistry of nitrogen mobilization: purine ring catabolism. Trends Plant Sci. 16, 381–387. 10.1016/j.tplants.2011.03.012 21482173

[B84] Wolf S. Marani A. Rudich J. (1991). Effect of temperature on carbohydrate metabolism in potato plants. J. Exp. Bot. 42, 619–625. 10.1093/jxb/42.5.619

[B85] Yalcinkaya T. Uzilday B. Ozgur R. Turkan I. Mano J. (2019). Lipid peroxidation-derived reactive carbonyl species (RCS): their interaction with ROS and cellular redox during environmental stresses. Env. Exp. Bot. 165, 139–149. 10.1016/j.envexpbot.2019.06.004

[B86] You J. Chan Z. (2015). ROS regulation during abiotic stress responses in crop plants. Front. Plant Sci. 6, 1092. 10.3389/fpls.2015.01092 26697045 PMC4672674

[B87] Zandalinas S. I. Mittler R. Balfagón D. Arbona V. Gómez-Cadenas A. (2018). Plant adaptations to the combination of drought and high temperatures. Physiol. Plant 162, 2–12. 10.1111/ppl.12540 28042678

[B88] Zhang S. Xu X. Sun Y. Zhang J. Li C. (2017). Influence of drought hardening on the resistance physiology of potato seedlings under drought stress. J. Integr. Ag. 16, 60345–60347. 10.1016/S2095-3119(17)61758-1

[B89] Zhao H. Wu D. Kong F. Lin K. Zhang H. Li G. (2017). The Arabidopsis thaliana nuclear factor Y transcription factors. Front. Plant Sci. 7, 2045. 10.3389/fpls.2016.02045 28119722 PMC5222873

