# Supplementary material for: Physiological, Biochemical, and Transcriptional Responses to Single and Combined Abiotic Stress in Stress-Tolerant and Stress-Sensitive Potato Genotypes
Source: Front Plant Sci. 2020 Feb 27;11:169. doi: 10.3389/fpls.2020.00169 (PMC7058966; doi:10.3389/fpls.2020.00169)
Supplement: Supplementary file 1 [file DataSheet_1.pdf]

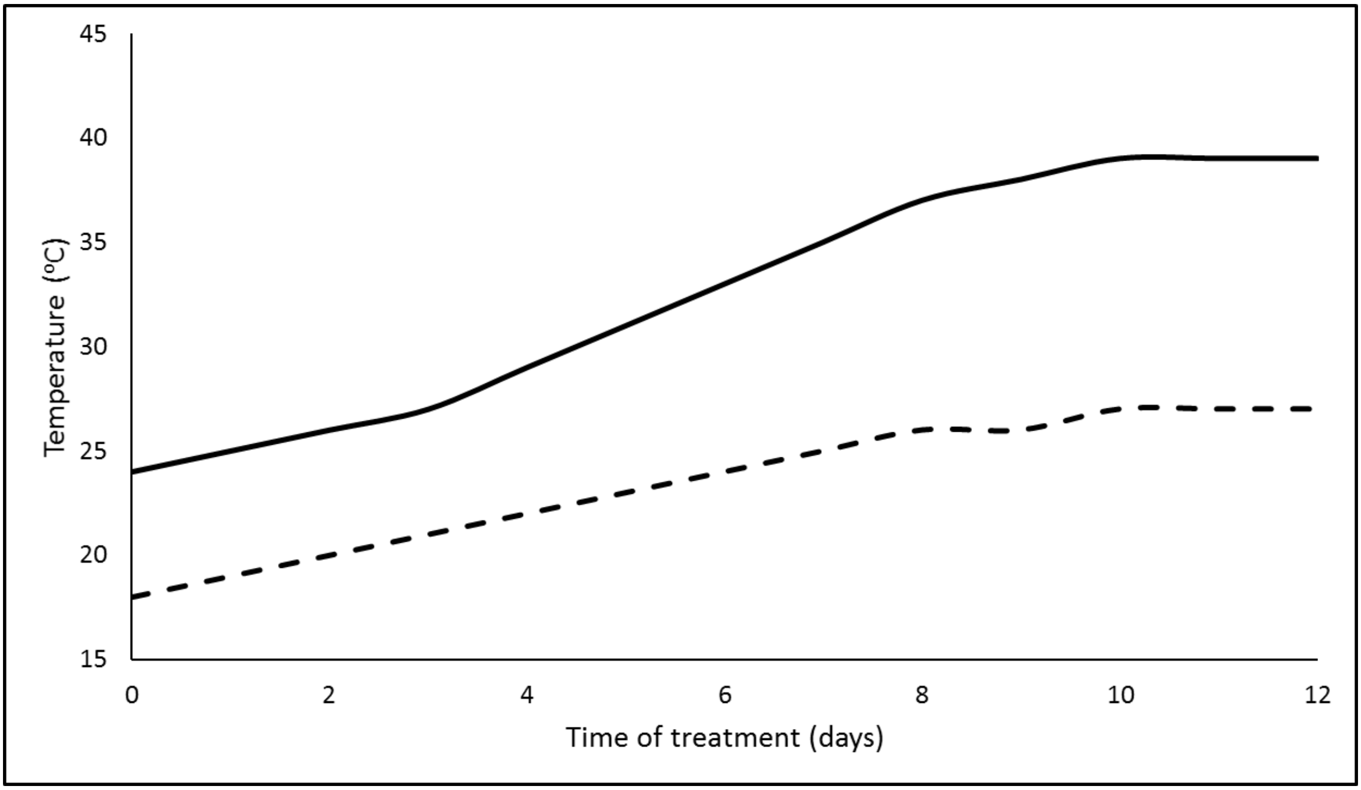

Supplementary Figure S1. Profile of temperature stress treatments. The figure illustrates changes in temperature profile during the day (▬) or night (▬ ▬) over the course of heat or heat and drought stress treatment.

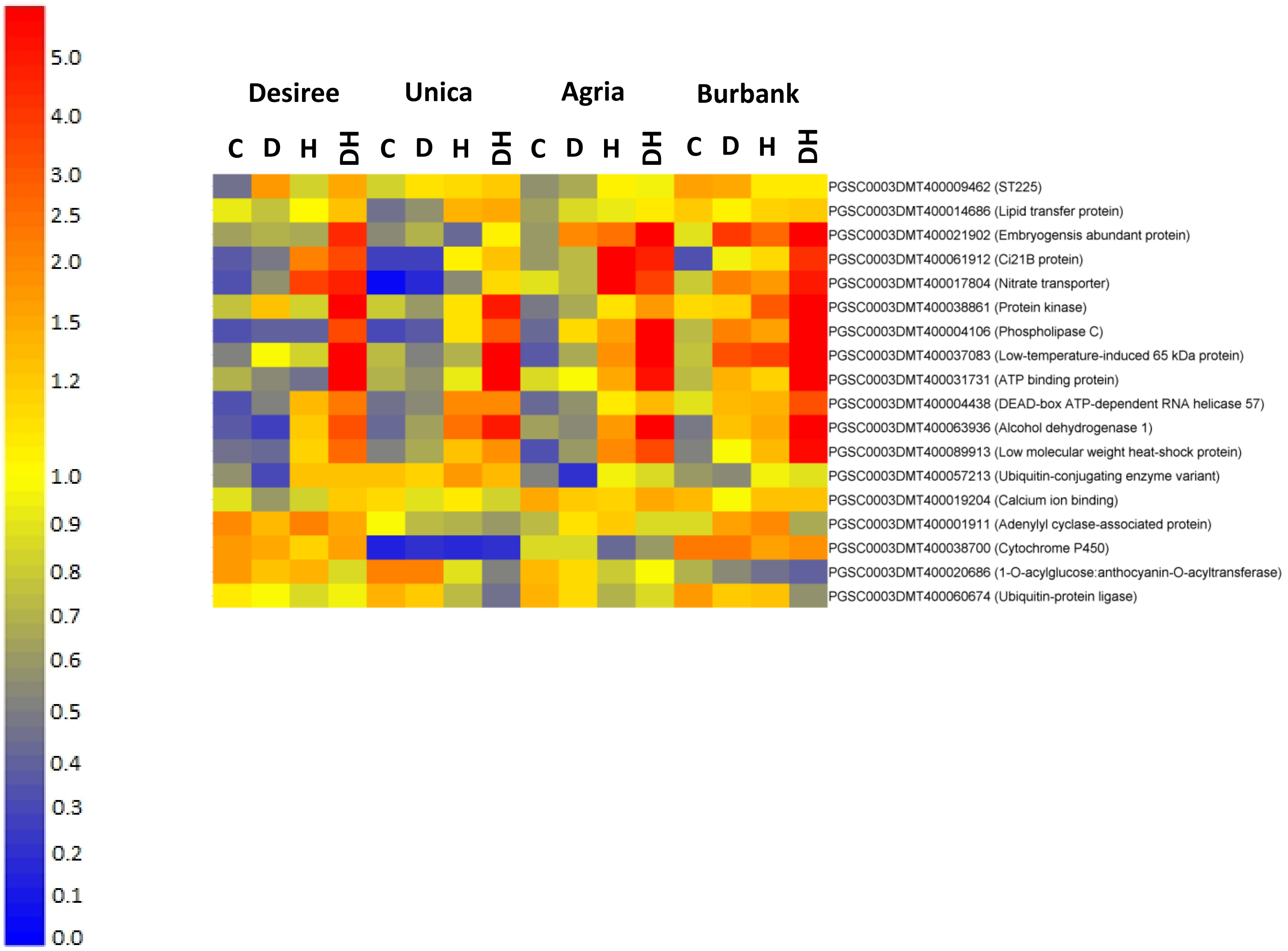

Supplementary Figure S2. Heatmap of differentially expressed transcripts previously identified as responding to drought (Pieczynski et al., 2018). Column labels indicate genotype and treatment (C, control; D, drought; H, heat; DH, drought and heat). Row labels indicate transcript accession number and description. Transcripts were clustered using GeneSpring and mean relative abundance of three independent biological replicates is indicated according to the scale bar shown.

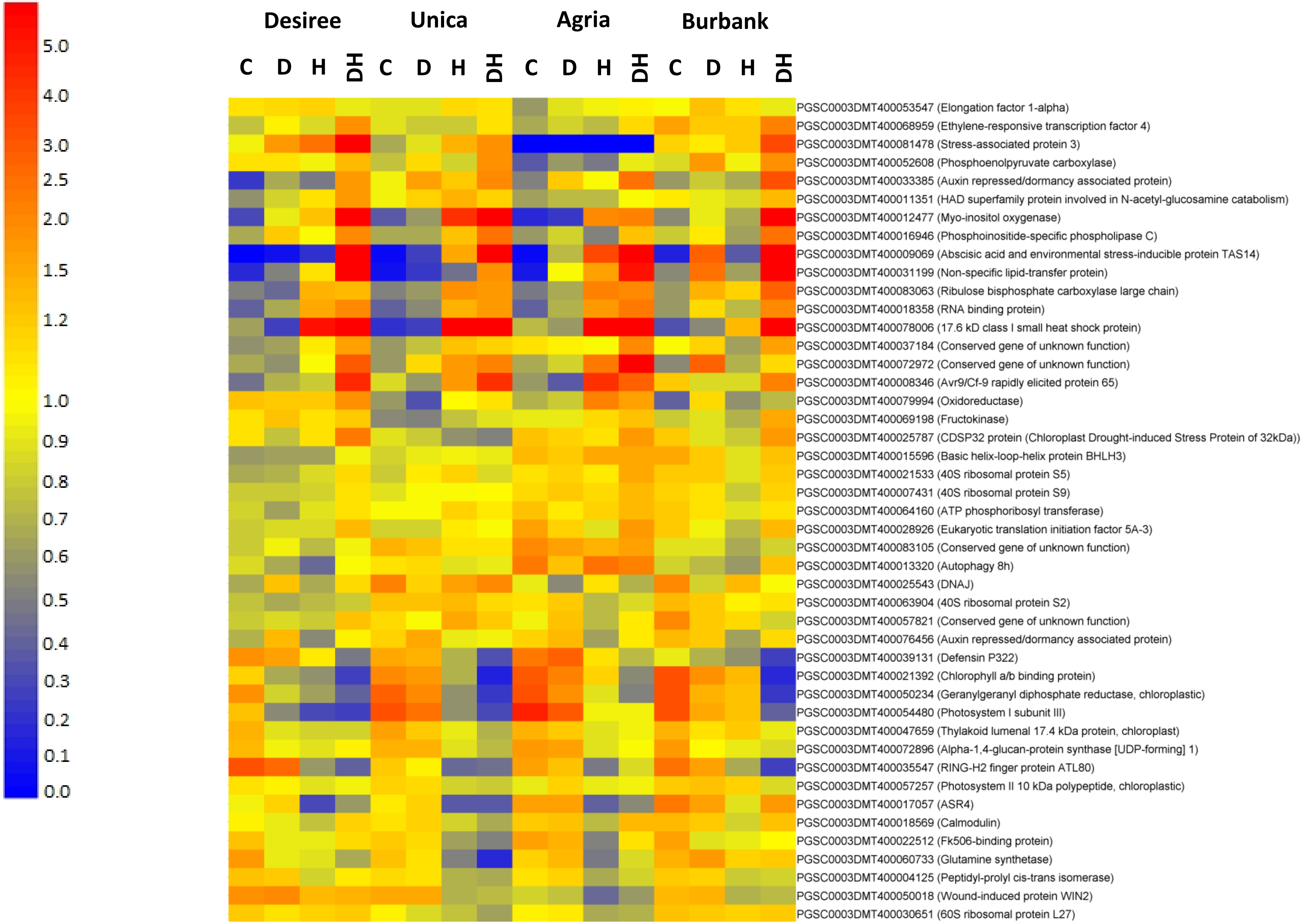

Supplementary Figure S3. Heatmap of differentially expressed transcripts previously identified as responding to heat (Gangadhar et al., 2014). Column labels indicate genotype and treatment (C, control; D, drought; H, heat; DH, drought and heat). Row labels indicate transcript accession number and description. Transcripts were clustered using GeneSpring and mean relative abundance of three independent biological replicates is indicated according to the scale bar shown.

Carbohydrates

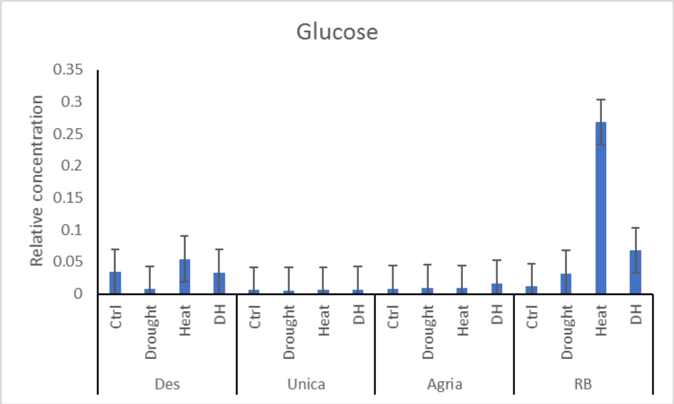

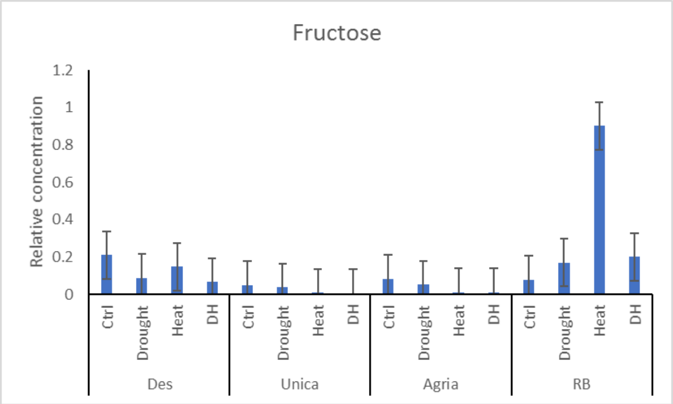

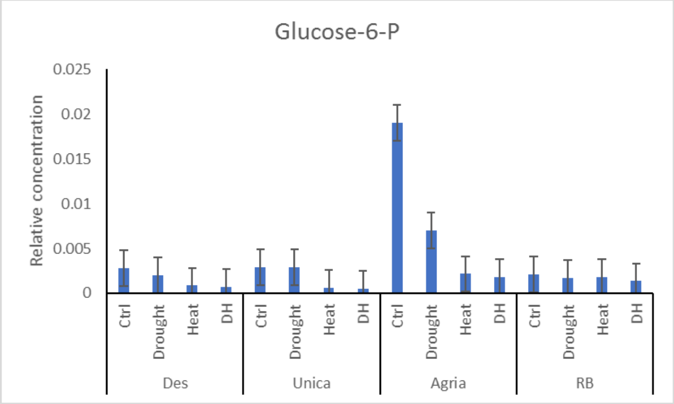

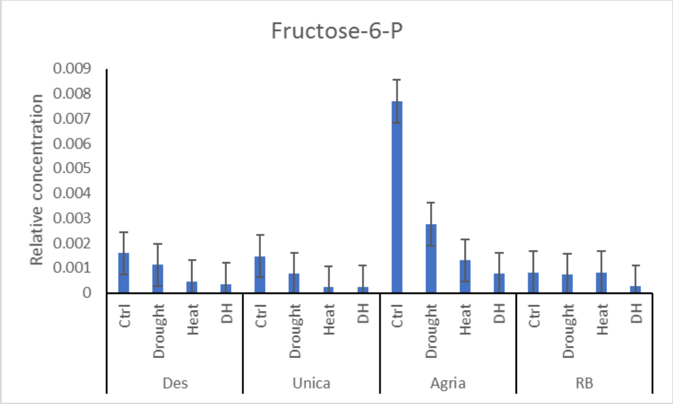

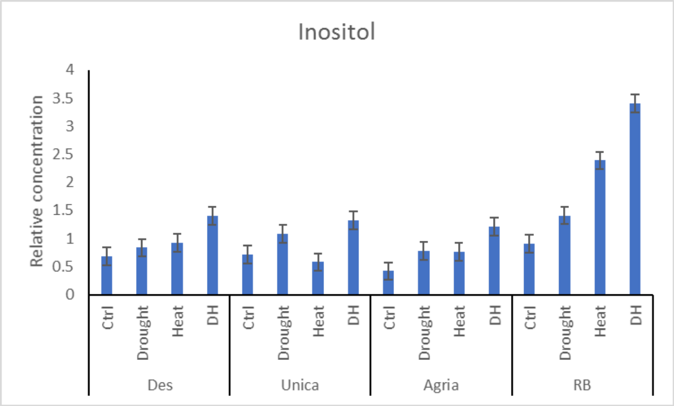

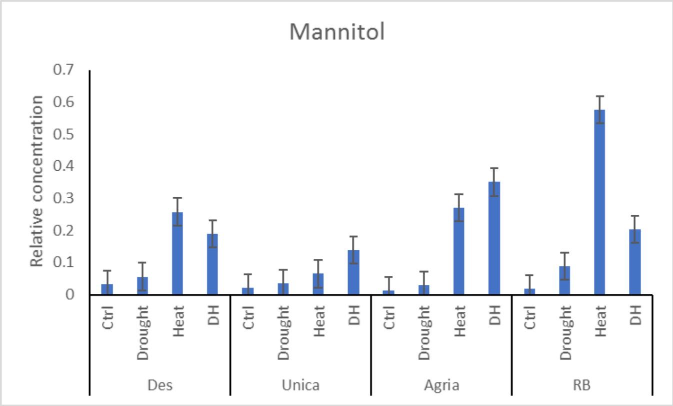

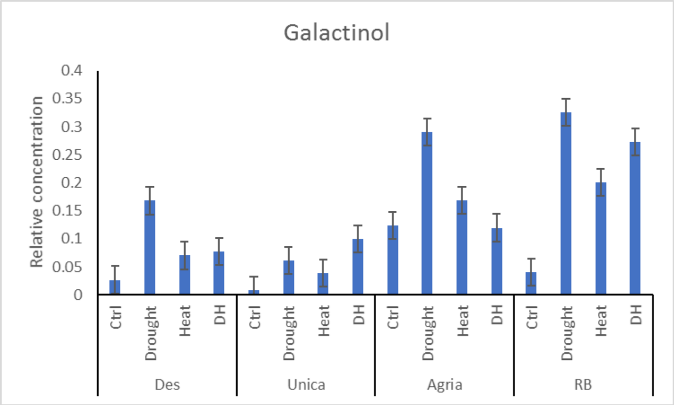

Organic acids

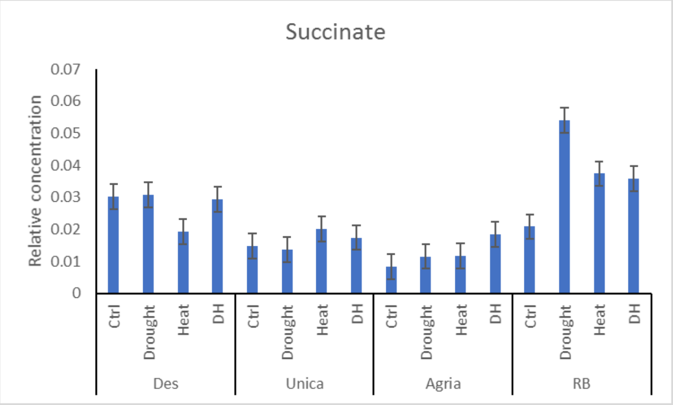

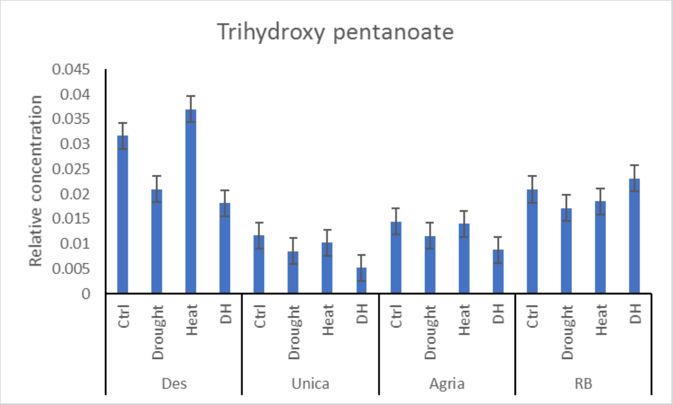

Amino acids and other amines

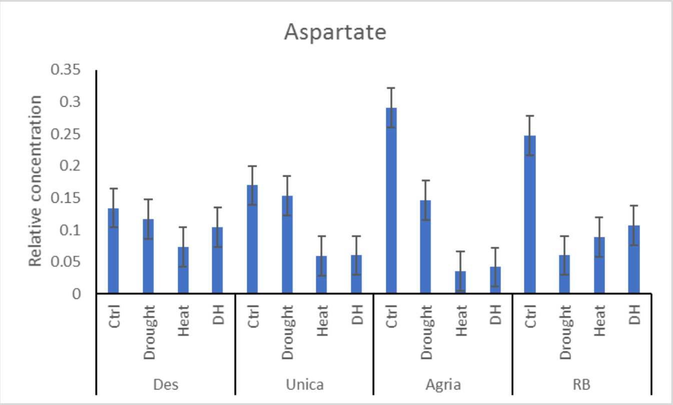

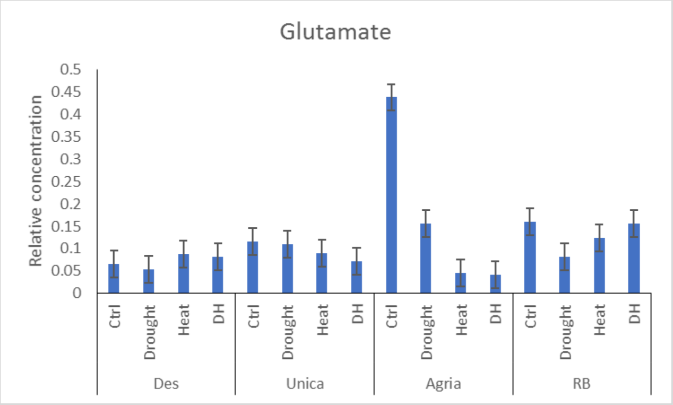

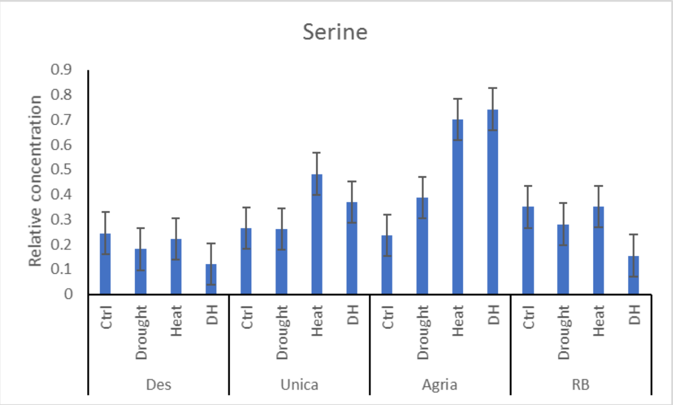

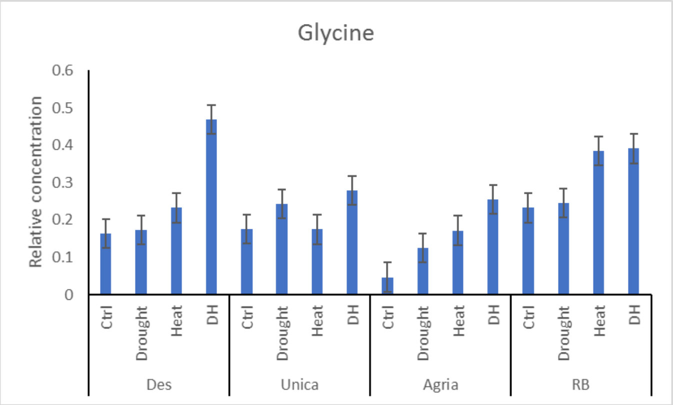

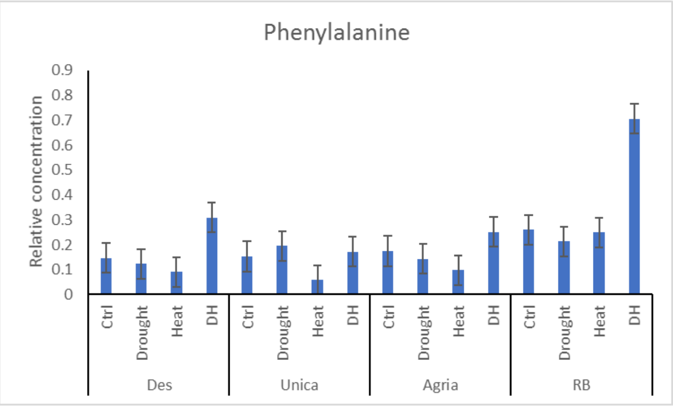

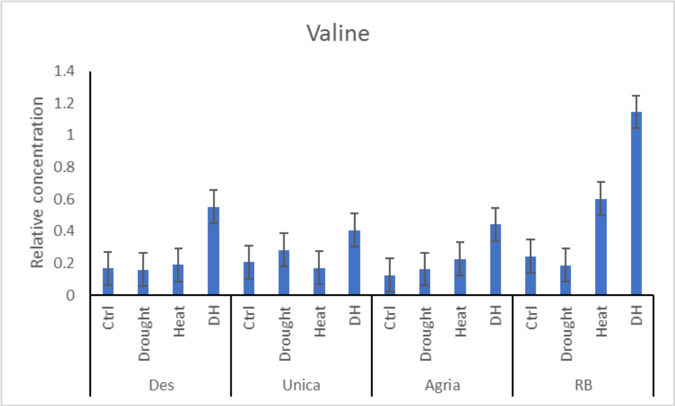

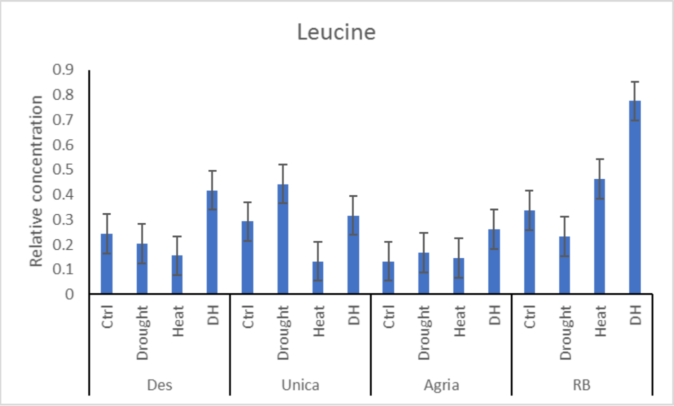

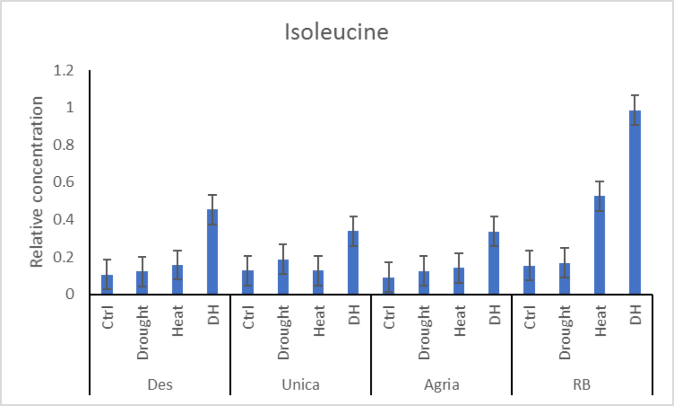

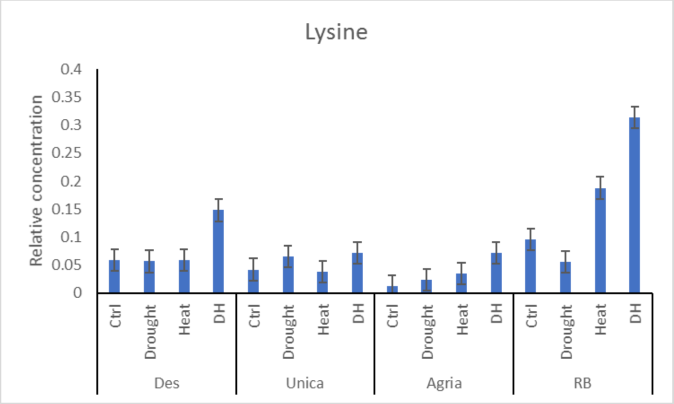

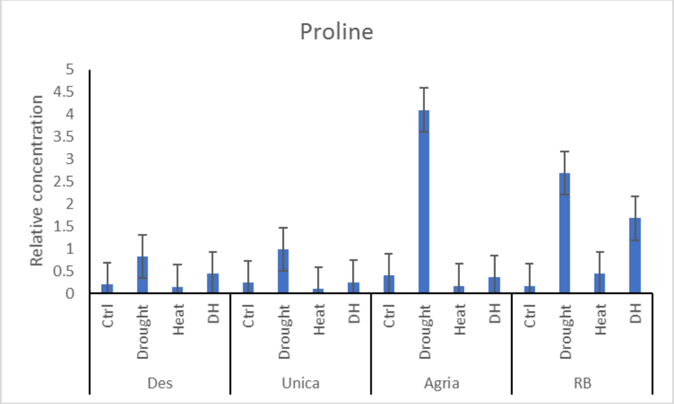

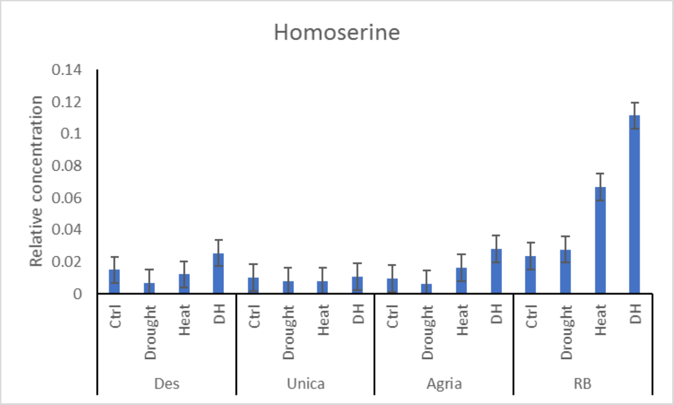

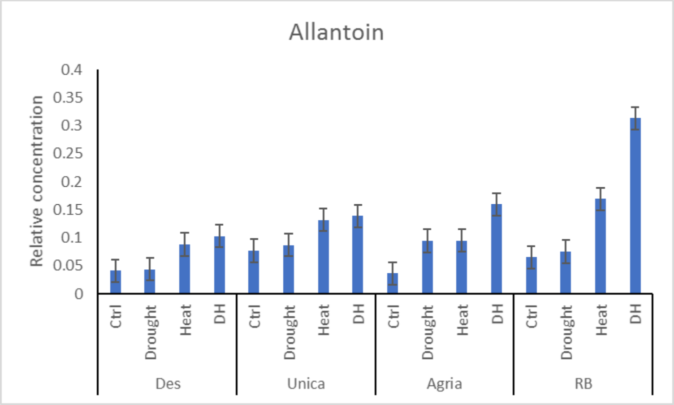

Fatty acids and alcohols

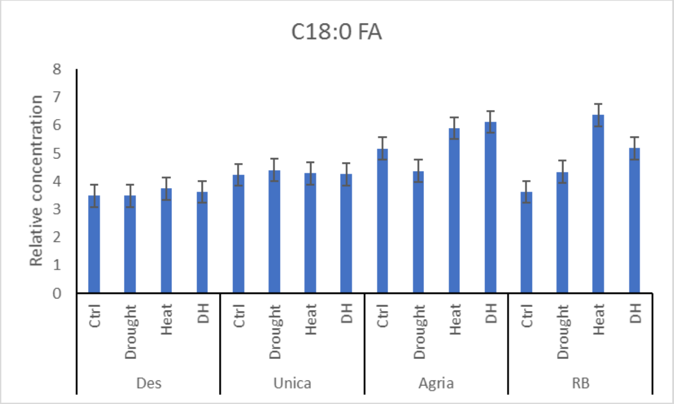

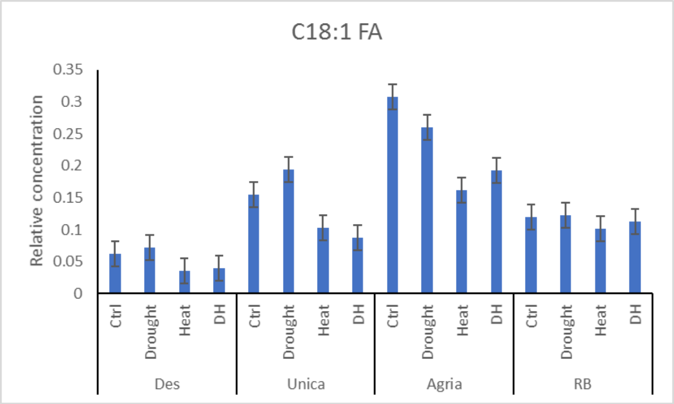

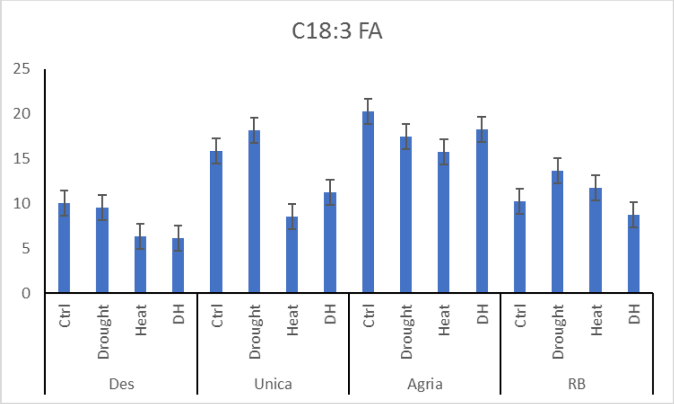

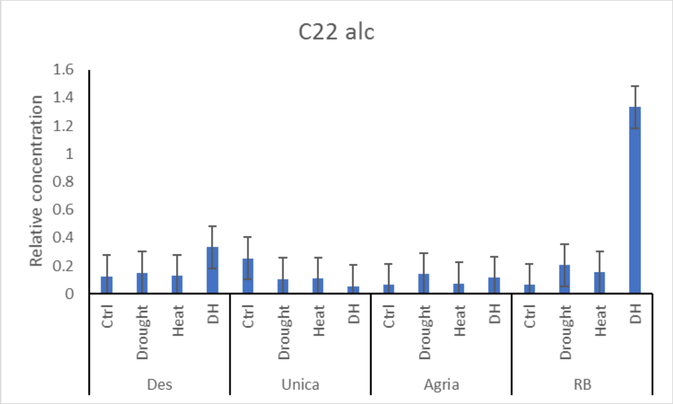

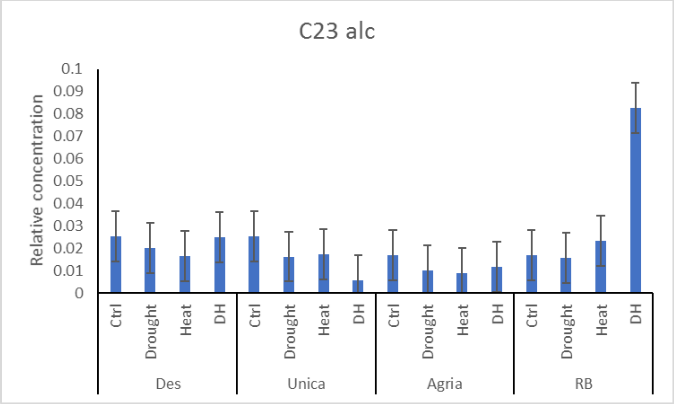

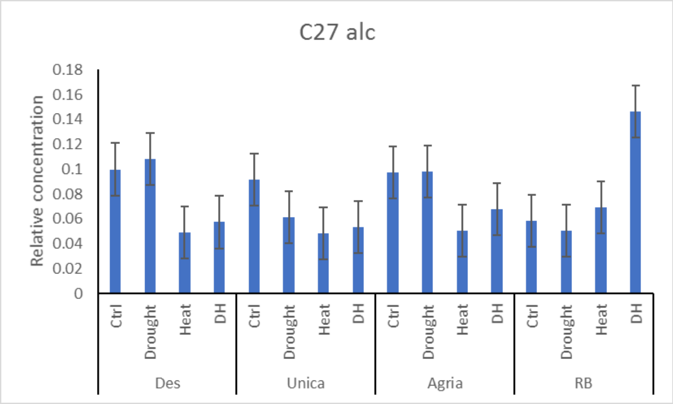

Unknown compounds

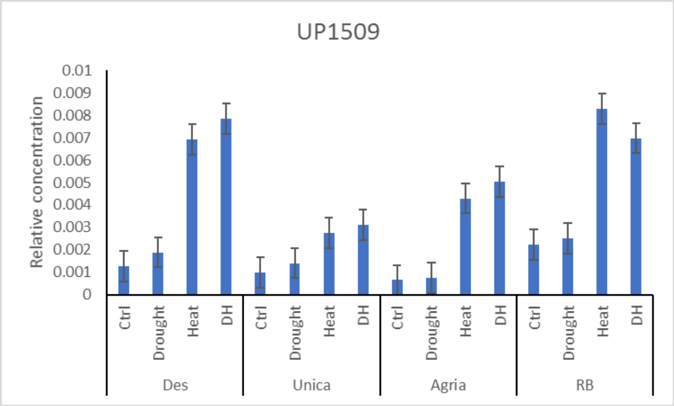

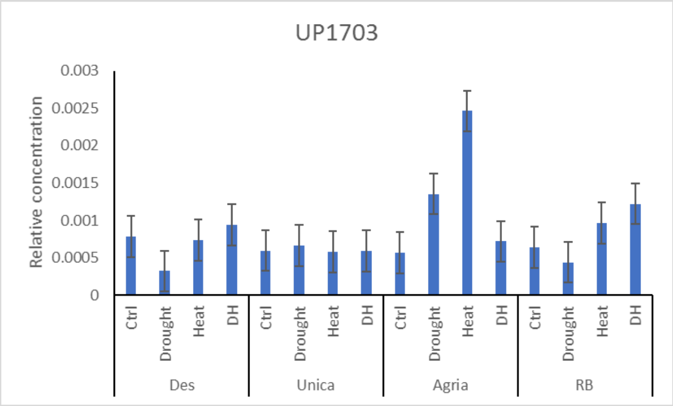

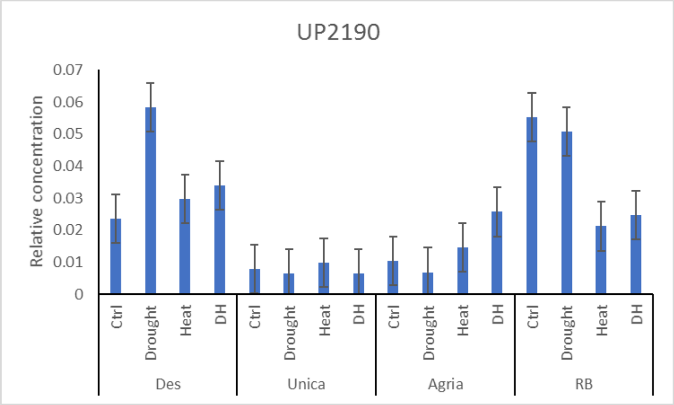

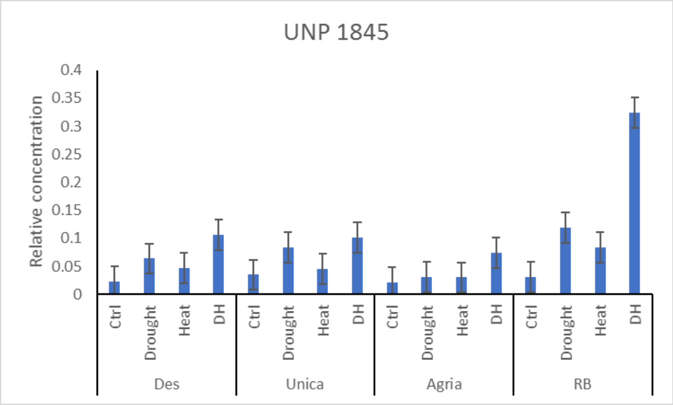

Supplementary Figure S4. Relative concentration of metabolites exhibiting a significant ($P<0.05$) genotype by metabolite interaction in their abundance in potato leaves. Columns illustrate the mean concentration relative to the appropriate internal standard and bars the overall standard error as estimated using ANNOVA. Fatty acids (FA) are described according to their chain length and number of double bonds (e.g. oleic acid is represented as C18:1 FA). Fatty alcohols are described according to their chain length followed by the abbreviation alc. Unknowns are listed as polar (UP) or non-polar (NP) followed by the mass spectrometry scan number of their peak apex.
